# Synoptic-to-interannual drivers of humid heat variability in equatorial Southeast Asia

**DOI:** 10.1038/s41612-026-01451-3

**Published:** 2026-06-09

**Authors:** Anistia M. Hidayat, Cathryn E. Birch, Lawrence S. Jackson, Juliane Schwendike, Claudio Sanchez, Adrian J. Matthews

**Affiliations:** 1 School of Earth and Environment, University of Leeds, Leeds, UK; 2 Meteorological, Climatological, and Geophysical Agency (BMKG), Jakarta, Indonesia; 3 Met Office, Exeter, UK; 4 School of Environmental Sciences, University of East Anglia, Norwich, UK

**Keywords:** Climate sciences, Natural hazards

## Abstract

As the Earth continues to warm, humid heat extremes (HHEs) have emerged as a widely recognised threat to human health in equatorial Southeast Asia (SEA). While most studies have focused on climate trends, this study presents a comprehensive analysis of the synoptic and large-scale drivers of HHEs. On daily timescales, both the Madden-Julian Oscillation (MJO) and Kelvin waves are the leading modes of HHE variability. HHE risk increases by 1.2–1.4x during the dry-to-wet transition of the MJO phases, predominantly driven by increased near-surface specific humidity preceding the peak rainfall anomaly in phase 2 and increased shortwave radiation due to reduced cloud cover in phase 8. HHE risk increases by 1.3–2.0x during the dry phase of equatorial Kelvin waves, which drives subsidence and increased shortwave warming. On interannual timescales, El Niño is the leading driver, under which HHE risk increases 3 – 5x. Despite the limited overlap (19%) between wet- and dry-bulb temperature extremes, the differences in their temperature and humidity conditions, and their drivers, are small. This understanding lays the groundwork for short-range to seasonal forecasts, which are a crucial component of much-needed heat early warning systems.

## Introduction

The year 2024 is currently the warmest year on record^1^, rapidly surpassing the previous record set in 2023^2^. Across the globe, heat extremes are increasing in frequency, duration, and intensity due to climate change^3–7^. Equatorial SEA has emerged as one of the most critical global hotspots for humid heat extremes (HHEs), primarily because the region is densely populated and climatologically very humid. High ambient humidity impairs evaporative cooling, a mechanism responsible for over 75% of heat dissipation from the human body^8^, which defines the unique threat posed by HHEs. Observational trends and projections consistently indicate that HHEs in SEA have intensified in recent decades and are expected to intensify further in the near future^8–10^. Record-breaking heat events occurred across SEA in April and May 2023, accompanied by severe health impacts, increased wildfires, and reduced agricultural productivity^11^. However, meteorological drivers of HHE^12^ variability across equatorial SEA remain poorly understood, particularly at the daily timescales relevant for extreme heat early warning systems.

Several large-scale atmospheric drivers are known to modulate heat extremes variability. El Niño events are associated with increases in the frequency^6^ and spatial extent of heat extremes, particularly in tropical regions^13–15^. Positive phases of the Indian Ocean Dipole (IOD) have been linked to intensified heat extremes in mid-latitude regions^16,17^, yet their impact on equatorial SEA is not well understood. The Madden–Julian Oscillation (MJO) also affects regional weather, with its active phases capable of doubling the likelihood of heat extremes over India^18^, yet its role in equatorial SEA remains largely unexplored. In equatorial SEA, synoptic features like Cross-Equatorial Northerly Surges (CENS) influence regional moisture transport^19,20^, and may also have potential role in preconditioning the environment for heat extremes.

Midlatitude atmospheric dynamics, particularly Rossby wave trains, are well documented for generating persistent so-called “heat domes” during heat extremes^21,22^. Equatorial Kelvin waves, westward-propagating mixed Rossby–gravity (WMRG) waves, and equatorial Rossby waves modulate daily weather variability over equatorial SEA^11,12,23–27^. While most studies have focused primarily on their influence on rainfall extremes during the convective wave phases^23,28^, Lyu et al.^11^ point to a role for equatorial Rossby and Kelvin waves in the unprecedented April-May 2023 SEA heat extremes.

Previous studies^11,13,15,18,29^ typically evaluate heat extremes drivers in isolation, or for a single case study, with limited quantification of their relative importance or of how interactions among tropical modes, including ENSO, modulates regional heat extreme risk. Moreover, much of the existing literature^14,30–33^ emphasises monthly or seasonal mean temperature anomalies, with comparatively limited attention to the daily-scale processes governing heat extremes specifically in equatorial SEA. In this region, there is also no consensus in the literature on whether humid and dry heat extremes should be treated within a common framework or considered as dynamically distinct phenomena. This study addresses these gaps by establishing the first quantitative relative-risk framework for equatorial SEA heat extremes. First, we analyse the dynamic and thermodynamic characteristics of HHEs over the last 32 years. Second, we quantify the relative contribution of the MJO and equatorial waves on daily to intraseasonal HHE variability and of ENSO and IOD on interannual variability. Third, we compare the characteristics of heat extremes defined using both wet- and dry-bulb temperature to determine whether they should be considered as the same or separate events. Insights from this study will lead to improved short-range to seasonal forecasting guidance on which metric to forecast and an evidence base for the development of regional early warning systems and climate risk management strategies.

## Results

### Characteristics of heat extremes in equatorial SEA

**Fig. 1:**
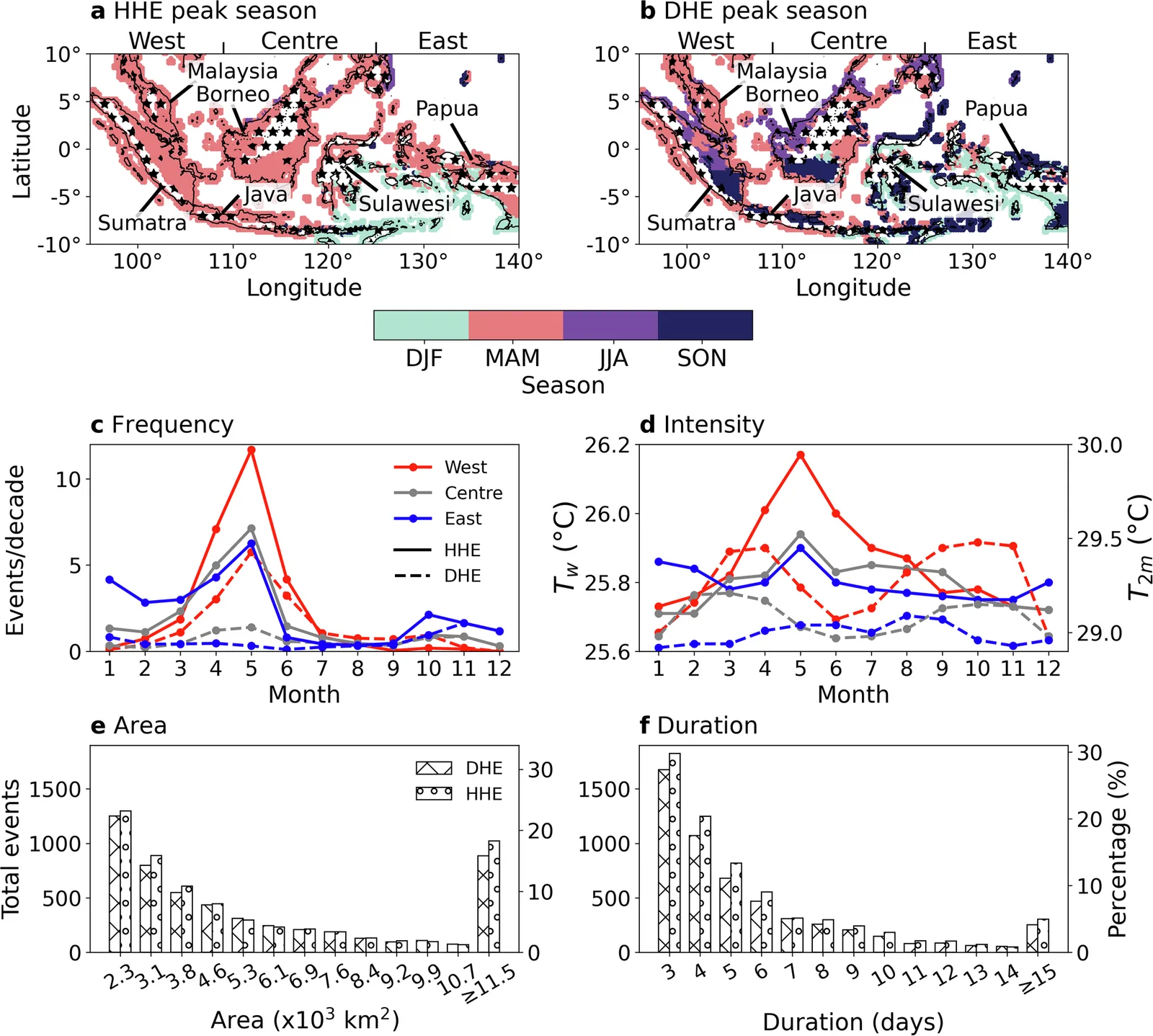
Characteristics of heat extremes across equatorial SEA. **a** Peak HHE season, defined as the three-month period with the highest number of events per land grid cell. The West (95°-109°E), Centre (109°-125°E), and East regions (125°-140°E) are defined by longitudinal boundaries, indicated by the labels and axis ticks at the top of the plot. Stars indicate land areas exceeding 500 meters elevation above mean sea level. **b** As in panel (**a**), but for DHE. **c** Total number of HHEs and DHEs per land grid cell per decade. Solid lines indicate HHEs and dashed lines indicate DHEs. **d** As in panel (**c**) but for daily mean of *T*_*w*_ (HHE) and *T*_*2m*_ (DHE) on the first day of each heat extreme event. **e** Spatial extent of heat extreme events (HHE: circle hatching, DHE: cross hatching). **f** As in panel (**e**) but for duration. All panels are derived from events identified over the period 1993–2024.

HHEs are diagnosed using 32 years of ERA5^34^ daily mean wet-bulb temperature (*T*_*w*_), which must be above the local 95th percentile and an absolute value of 25.5 °C for at least 3 consecutive days and cover an area of approximately 2300 km² (see Methods). HHE occurrence peaks in March – May (MAM; Fig. 1a), with 82% of land grid cells in equatorial SEA experiencing their highest HHE frequency in this season. The timing of the HHE peak season aligns with the maxima in the annual cycle of the near-surface temperature and specific humidity (Fig. S1). In contrast, 16% of SEA, primarily in the East, experiences peak HHEs during December–February (DJF). The East is characterised by a monomodal rainfall peak in June-August (JJA) and weak influence from the Asian–Australian monsoon and the ITCZ^35^. Consequently, while much of equatorial SEA experiences its wet season from November to March (NDJFM), the East receives the least precipitation during this period^35^.

The West experiences substantially higher frequencies (Fig. 1c) and intensities (Fig. 1d) of HHEs than the Centre and East regions, with at least one event every year on average. One contributing factor is the regional topography where approximately 66% of the West region lies below 500 meters (Fig. 1a), where temperatures are warmer due to the atmospheric lapse rate, compared to 50% and 46% of land below 500 meters in the Centre and East regions, respectively. The fragmented, archipelagic geography of equatorial SEA countries limits the spatial footprint of HHEs, leading to smaller-scale events than those occurring over the large, contiguous landmasses characteristic of mid- and high-latitudes^36^. Across the three subregions, 23.3% of events cover only 2300 km² (Fig. 1e), whilst 18.3% cover more than or equal to 11,532 km². Approximately half of HHE events (50%) persist for a duration of three or four days (Fig. 1f).

While this study focuses on HHEs, we also consider heat extremes identified using the same event criteria but based on an absolute dry-bulb temperature (*T*_*2m*_) threshold of 28.5° °C (hereafter referred to as dry heat extremes, DHE; see Methods). Like HHEs, most of equatorial SEA record their highest DHE frequency in MAM (45%), although in some regions (26%) their peak season is observed in September to November (SON, Fig. 1b). During the MAM peak season, the West region consistently shows a higher frequency of DHEs, with at least one event every two years, than the Centre and East regions (Fig. 1c). DHE intensities are also highest in the West, with daily mean *T*_*2m*_ reaching 29.2°–29.5 °C and a secondary peak of 29.4°–29.5 °C in SON (Fig. 1d). The Centre region shows a similar bimodal seasonal pattern but with lower values (29.1°–29.2 °C), whereas the East region has a weaker seasonal cycle, with mean *T*_*2m*_ generally below 29.1 °C. The higher frequency and intensity of both HHEs and DHEs in the West are consistent with recent climate model projections^10^.

Besides sharing a primary peak season in MAM (Fig. 1a, b), equatorial SEA HHEs and DHEs typically have similar spatial extents and durations (Fig. 1e, f). To quantify the degree of synchronisation between these two types of heat extremes across equatorial SEA during MAM, the Jaccard index^37^ is utilised. This metric evaluates the intersection of two binary datasets relative to their union across a three-dimensional domain (time x latitude x longitude). Across equatorial SEA, the spatiotemporal overlap between HHEs and DHEs during MAM is up to 19%, although this proportion is sensitive to our chosen lower thresholds (25.5 °C for HHEs and 28.5 °C for DHEs).

**Fig. 2:**
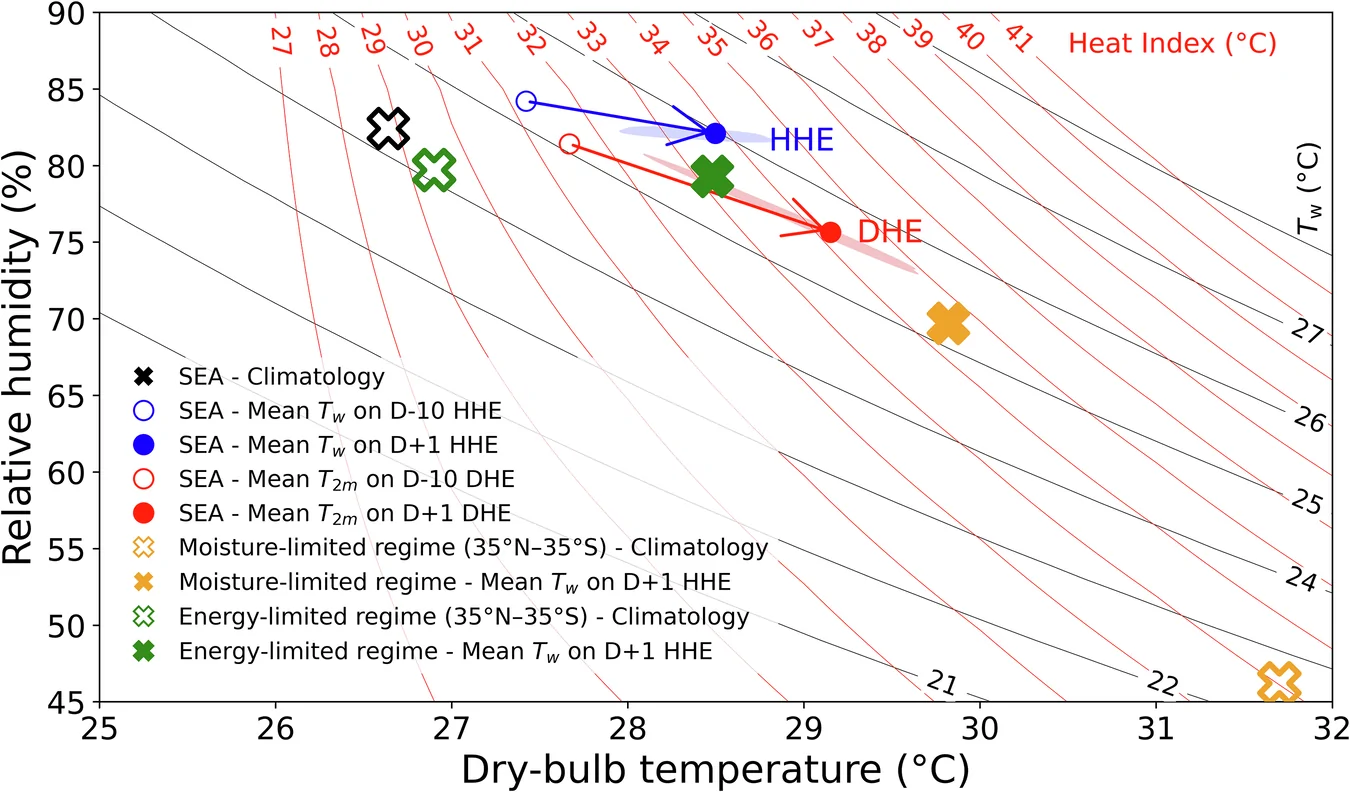
Daily mean temperature and humidity conditions during heat extreme events in the equatorial SEA averaged over the West, Centre, and East and across the global (sub)tropics. Blue and red open circles depict conditions 10 days before (D-10) the first day of HHEs and DHEs (i.e. D0, onset day), respectively. Arrows link these points to the mean conditions on the second day of each HHE (blue filled circle) and DHE (red filled circle), when *T*_*w*_ and *T*_*2m*_ typically reach their maximum (i.e. 2nd day, D + 1). Shaded regions represent the distribution during the second day of HHE and DHE using Gaussian Kernel density estimation, encompassing 95% of the events. The unfilled black cross represents the equatorial SEA climatology, averaged over all months. Climatological values (green and yellow open cross symbols) and values on the second day of HHEs (solid cross symbols) averaged across all moisture- (orange) and energy- limited (green) regions are average globally between 35°N and 35°S, taken from Jackson et al.^39^.

To determine if HHE and DHE should be considered as separate phenomena or not, it is instructive to plot the events on a relative humidity (RH) vs *T*_*2m*_ diagram, where higher *T*_*w*_ and Heat Index (HI, a metric combining T_2m_ and RH) are towards the top right (Fig. 2). The climatology in equatorial SEA (black cross) is high in humidity but only moderately high in temperature. Ten days before the commencement of HHE and DHE events (unfilled blue and red circles), their *T*_*2m*_ values are already almost 1 °C warmer than climatology. During both types of events, *T*_*w*_ is on average 26 °C and HI is 34 °C, exceeding the ‘Extreme Caution’ threshold (HI > 33 °C)^38^. It is important to note that these classifications are based on daily mean *T*_*w*_, which captures prolonged exposure but may underestimate peak stress levels. Using daily maximum *T*_*w*_ would shift more events into more severe HI categories.

Equatorial SEA heat extremes are now examined within a broader tropical and subtropical context (35°N – 35°S) using data from Jackson et al.^39^, who diagnosed their HHEs in the same way as this study, albeit with a lower absolute threshold of *T*_*w*_ = 24 °C. In their approach, events are grouped by whether the region is moisture-limited with higher *T*_*2m*_ and lower humidity, or energy-limited with lower *T*_*2m*_ and higher humidity during the season of extreme heat at each location (see their Fig. 5). The difference between the climatologies (unfilled green and yellow crosses, Fig. 2) and the HHE conditions (filled crosses) in the moisture and energy-limited regions in Jackson et al.^39^ is stark and equatorial SEA’s climatology closely aligns with that of the energy-limited regime. Moreover, the equatorial SEA HHE and DHE events have *T*_*2m*_ and RH characteristics that are more similar to these energy-limited HHE events (filled green cross) than their moisture-limited HHE events (filled yellow cross).

To further assess the difference between HHEs and DHEs in equatorial SEA, we quantify the relative contribution of *T*_*2m*_ and 2m specific humidity (*q*_*2m*_) to *T*_*w*_ using the method of Rocuet et al.^40^ (see Methods). While DHEs are identified based on *T*_*2m*_ anomalies, their decomposition is evaluated using *T*_*w*_ during DHE days to maintain a consistent metric for comparison with HHEs. On non-heat-extreme days, *T*_*w*_ is primarily moisture-controlled (68.4%), with the remaining 31.6% from *T*_*2m*_. During HHEs, this moisture dependence is amplified to 73.2% (SE ± 0.02, SD ± 10.8), whereas during DHEs it decreases to 65.9% (SE ± 0.31, SD ± 25.2). The small standard error values across both categories confirm that these mean dependencies are statistically robust and well-defined. The larger standard deviation for DHEs reveals a higher degree of event-to-event heterogeneity. This suggests that while the moisture contribution is lower on average during DHEs, the high internal variability allows for certain DHE days to exhibit moisture dependencies comparable to those observed in HHEs.

These results suggest that equatorial SEA HHE and DHE events, as defined in this paper, are fairly similar in their temperature and humidity conditions when put into the global context, and should not necessarily be considered as different phenomena, despite not often overlapping precisely in time and space. In the following sections, we focus our analysis on HHEs, returning to the differences between HHE and DHE drivers in the final section of the results.

### Physical drivers of humid heat extremes

**Fig. 3:**
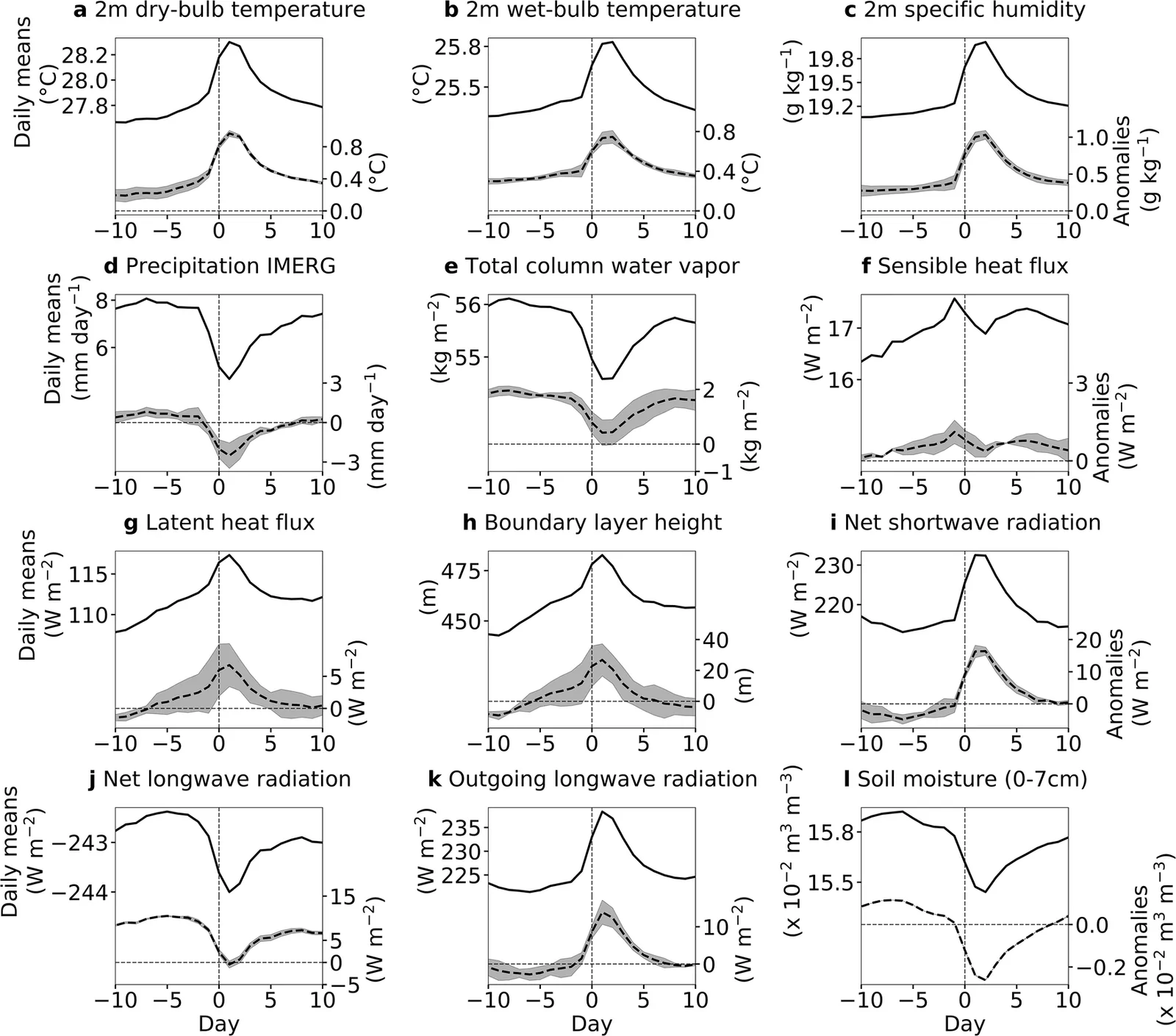
Composite time series during HHEs, averaged over the West, Centre, and East regions. The analysis is based on ERA5 reanalysis data, except precipitation, which is from GPM-IMERG. Daily mean values and anomalies are composited on day 0 (i.e. onset day) of the events. Panels show daily mean values (solid lines) and anomalies (dashed lines) of (**a**) 2m dry-bulb temperature, (**b**) 2m wet-bulb temperature, (**c**) 2m specific humidity, (**d**) precipitation IMERG, (**e**) total column water vapor, (**f**) sensible heat flux, (**g**) latent heat flux, (**h**) boundary layer height, (**i**) net shortwave radiation, (**j**) net longwave radiation, (**k**) outgoing longwave radiation, and (**l**) soil moisture (0–7 cm) relative to the 1993–2024 daily climatology smoothed with a 15-day moving average, except panel (d) which covers 2001–2024. Shaded areas represent the 95% confidence intervals for the daily anomalies.

Figure 3 presents composite time series of the key surface and atmospheric variables, shown as absolute values and anomalies relative to a 15-day smoothed climatology (see Methods), centred on D0, defined as the first day, or onset day, of each HHE event. We average results over the West, Centre and East regions because there is little difference between the separate results (not shown). In the lead up to D0, the positive anomaly in *T*_*w*_ grows, driven by similar positive anomalies in both *T*_*2m*_ and *q*_*2m*_ (Fig. 3a–c). There is also a positive anomaly in total column water vapour, which goes to zero on D0, accompanied by a suppression of rainfall that starts on D-2 (Fig. 3d, e). This evolution during HHEs is typical of the energy-limited regions defined in Jackson et al.^39^, where heat extremes commonly follow periods of suppressed rainfall.

Changes in the sensible heat flux remain minimal throughout the event lifecycle (Fig. 3f). Instead, the surface energy partitioning strongly favours the latent heat flux (values of 110–115 W m^-2^, compared to sensible heat flux values of 16-17 W m^-2^). Anomalies in both heat fluxes are small ( < 5 W m^-2^), although a peak in latent heat flux during the events is evident (Fig. 3g). These surface conditions influence the overlying atmospheric boundary layer, primarily through a surplus in net radiation, which further offset evaporative moisture flux. The warming surface leads to a modest but distinct increase in boundary layer height, which experiences a positive anomaly of +23 m during the peak phase (Fig. 3h). Although not directly linked, the boundary layer growth could be partly driven by an increase in net surface radiation, indicated by a positive anomaly in incoming shortwave radiation ( + 13 W m^-2^, Fig. 3i), due to reduced cloud cover as indicated by a peak in outgoing longwave radiation (Fig. 3k).

At the same time, upwelling longwave radiation from the warming surface also increases by approximately 5 W m^-2^ (Fig. 3j), but this loss is partially moderated by the moist atmosphere. Even though there is a decrease in total column water vapour on D-2 to D + 2, the background atmospheric humidity, represented by specific humidity and total column water vapour, and soil moisture (Fig. 3l) over equatorial SEA remains relatively high, leaving substantial residual water vapour in the column and plenty of moisture for evaporation from the surface. This residual moisture acts as a radiative buffer by absorbing and re-emitting part of the outgoing longwave radiation. As a result, the longwave radiation anomaly decreases but remains near zero during the event peak. This condition limits what would otherwise be much stronger surface radiative warming and moderates the growth of the boundary layer. In all variables, the anomalies from climatology during events are relatively small compared to their absolute values.

The analysis suggests a plausible influence from larger-scale drivers on HHEs. Persistent anomalies at D − 10 and D + 10 suggest that HHEs evolve within a slowly varying large-scale background state, potentially associated with broader climate variability such as ENSO. In this context, the daily evolution shown in Fig. 3 likely develops on top of a pre-conditioned environment. This interaction between large-scale background states and local extremes is explored in the following sections.

### Atmospheric circulation during humid heat extremes

**Fig. 4:**
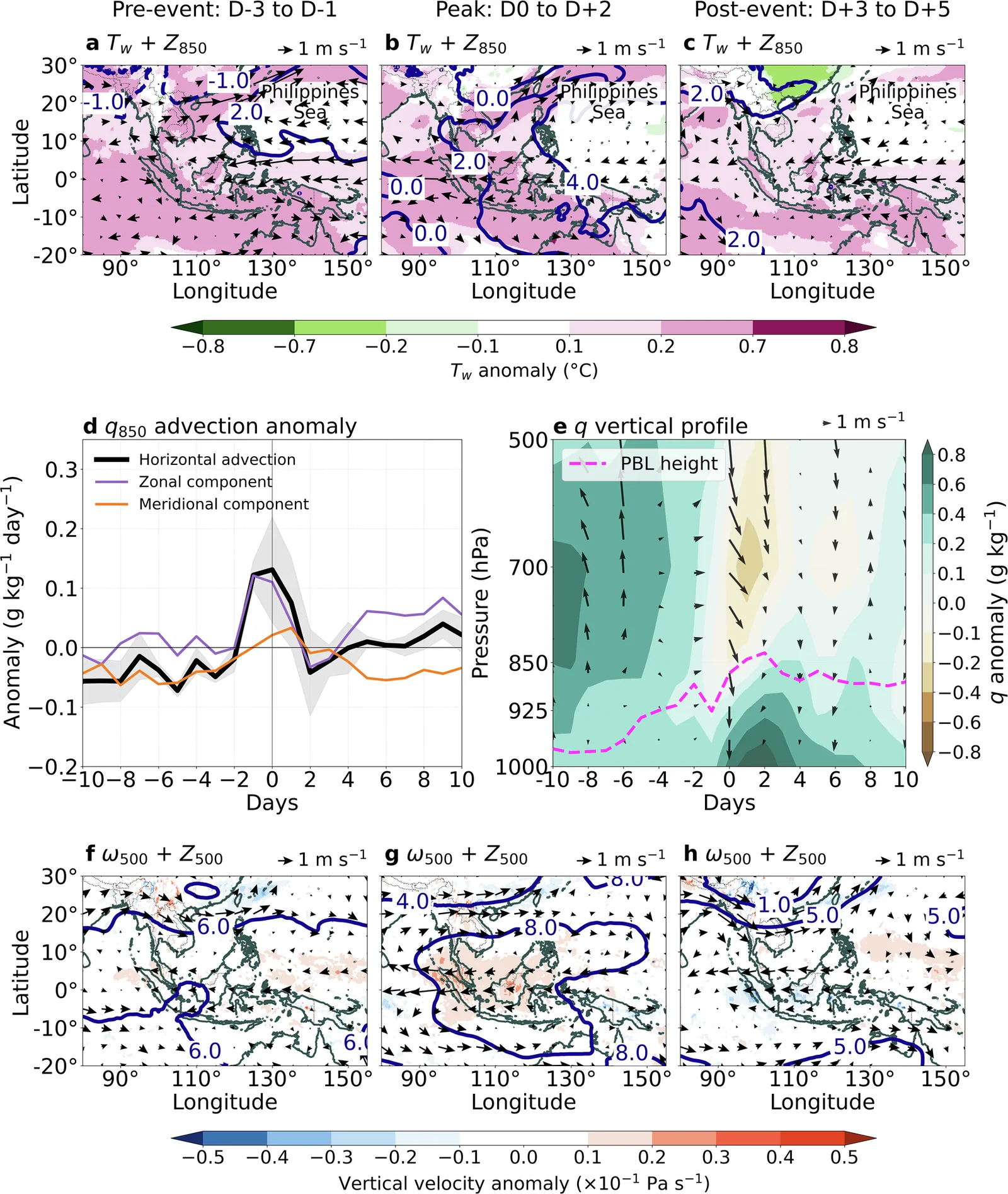
Anomaly composites of meteorological variables from ERA5 reanalysis during MAM HHEs in the West region. **a**, **b**, **c** Anomalies of *T*_*w*_ (°C; shading) during the pre-event (left), peak (middle), and post-event (right) periods, overlaid with anomalies of 850 hPa geopotential height (*Z*, contours) and wind (*UV*, vector). **d** Composite time series of horizontal specific humidity advection anomaly at 850 hPa (black line) and its corresponding decomposition into zonal (purple) and meridional (orange) components. Shaded areas denote 95% confidence intervals. **e** Time-pressure cross-section of anomalous specific humidity (g kg^-1^, shaded) and vertical-zonal wind vectors (m s^-1^, vector), with purple dashed-line indicates the daily maximum PBL height in hPa. **f**, **g**, **h** As in panel (**a**–**c**) but for anomalies of 500 hPa vertical velocity (*ω*, Pa s⁻¹; shading), geopotential height (m; contours), and wind (m s^-1^; vectors). Positive *ω* anomalies correspond to anomalous downward motion (subsidence), while negative anomalies indicate anomalous upward motion (ascent). Anomalies are calculated relative to local climatological daily means for the 1993–2024 period, smoothed with a 15-day moving average.

Atmospheric conditions are analysed during the pre-event (D-3 to D-1), peak (D0 to D + 2), and post-event (D + 3 to D + 5) periods in the West region (Fig. 4). On D-3 to D-1, a high-pressure anomaly develops over the Philippine Sea (Fig. 4a) and intensifies on D-2 to D0, forming a ridge across the equatorial SEA (Fig. 4b) that suppresses cloud formation. Throughout HHE evolution, anomalous easterly flow dominates 850 hPa (Fig. 4a–c). There is a positive anomaly in both *T*_*w*_ (Fig. 4a–c) and *q*_*2m*_ (Figs. S2a–c) across most of the domain, except near the high-pressure centre at 125°-150°E, 0°-20°N. Between D-1 to D + 2, the relatively homogeneous moisture field produces a weak spatial gradient, across which anomalous easterly winds drive minor positive horizontal moisture advection anomalies (0.07–0.13 g kg^-1^ day^-1^), dominated by the zonal component (Fig. 4d). By D + 3 to D + 5, the high-pressure anomaly weakens and easterly wind anomalies subside (Fig. 4c).

The 850 hPa high-pressure and easterly wind anomalies extend upward into the mid-troposphere (500 hPa, Fig. 4f–h). Anomalous subsidence at 500 hPa is present from D-3 to D-1 and intensifies during D0 to D + 2. The enhanced subsidence suppresses convection and cloud formation (Figs. 3d, k) which can drive surface warming via both shortwave and adiabatic heating. The resulting daytime surface heating favours the development of a deeper boundary layer (Figs. 3h, 4e). Under these conditions, the close temporal alignment between enhanced 850 hPa horizontal moisture advection (Fig. 4d) and increasing near-surface humidity (Figs. 4e, 3c) suggests vertical coupling where subsidence and boundary layer growth facilitate the entrainment of moist air from the lower troposphere into the near-surface air.

The vertical specific humidity (*q)* anomaly profile (Fig. 4e) further reveals a top-down moistening sequence at 850–700 hPa (D-10 to D-3), with horizontal moisture advection contributes to the development of an elevated moisture reservoir. During the first three days of HHEs (D0 to D + 2), intensified anomalous subsidence, along with the deepening of the planetary boundary layer (PBL), facilitates the vertical entrainment of this descending moist air. The increase in *q* at D + 2 likely reflects the cumulative effect of this coupling, where moisture is confined near the surface by a stabilising subsidence inversion aloft. On D + 3 to D + 5 (Fig. 4h), the mid-tropospheric high-pressure anomaly weakens and the intensity of subsidence diminishes in many areas. Broadly similar atmospheric circulation conditions occur across the Centre and East regions (Fig. S3), especially during D0-D + 2, however, in the East, the high-pressure anomalies are displaced further eastward, centred over Papua New Guinea.

The relative roles of advection, adiabatic heating, and diabatic heating are quantified through a low-level Lagrangian temperature budget analysis following Röthlisberger and Papritz^41^ (Figs. S4a–g and Supplementary Method A), which, despite assuming a dry atmosphere, is currently the most appropriate methodology available in the literature. Anomalies in all three terms start to appear 12 days prior to HHE onset. Consistent with a subtropical boundary-layer heat budget study^42^ that identified diabatic heating as a dominant factor, our budget analysis shows that diabatic processes dominate across 61.3% of locations in equatorial SEA, contributing approximately 0.6–1.0 °C to the temperature increase during the two days preceding HHE onset. A combination of diabatic and adiabatic heating dominates another 21.1% of the region. In the West region, the magnitude of the adiabatic term is only 14% of that of the diabatic contribution. The contribution of temperature advection is negative (cooling), although Fig. 4d shows that moisture advection remains critical, potentially supplying the humidity necessary to increase *T*_*w*_ and fuel the diabatic heating term.

Compared with mid- and high-latitude heat extremes^21,22,43^, where geopotential height anomalies could reach hundreds of meters at 500 hPa^44^, HHEs across equatorial SEA occur under weaker synoptic anomalies. This difference is partly a consequence of the weak tropical Coriolis force, which yields a large Rossby deformation radius^45^ and limits the amplitude of the geopotential anomalies by spreading them over broad spatial scales. Nonetheless, the occurrence of equatorial SEA HHEs under conditions of high-pressure and subsidence anomalies combined with enhanced surface solar radiation confirms that the underlying dynamics are fundamentally akin to those driving heat extremes at higher latitudes^21,46^.

### Role of local sea surface temperature

**Fig. 5:**
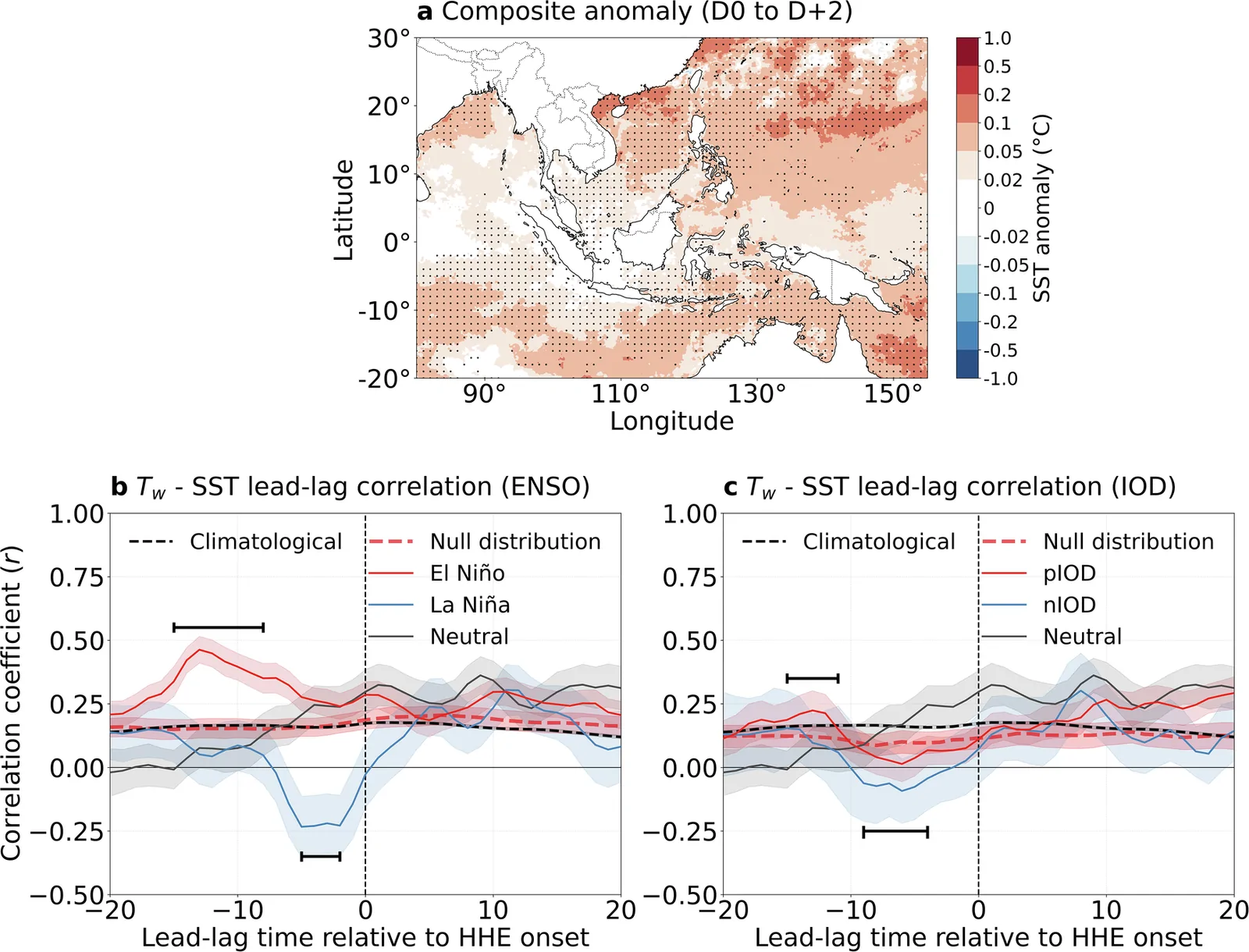
SST anomalies during HHEs across all three regions. **a** SST anomalies (°C, shading) during D0 – D + 2 relative to HHE onset. Stipplings marks the regions where anomalies are statistically significant at the 95% confidence level based on a two-tailed Welch’s t-test against non-HHE days in MAM season. **b** Lead-lag correlations between SST and *T*_*w*_ anomalies relative to HHE onset in three ENSO phases. **c** Same as (**b**), but for IOD. The horizontal black lines with vertical end caps indicate the window at which the correlation coefficient reaches its maximum departure from the null distribution. The black dashed lines represent the climatological correlation based on all MAM days. The red dashed lines indicate the mean of the null distribution, constructed by resampling event dates across different years within El Niño (**b**) and positive IOD (c, pIOD) years. Shaded areas in panel (**b**) and (**c**) denote 95% confidence intervals derived from 1000 bootstrap iterations. Anomalies are calculated relative to long-term daily MAM means over 1993–2024 and smoothed using a 15-day moving average. SST data are from European Space Agency’s Climate Change Initiative (ESA CCI).

Given that equatorial SEA is an archipelago of islands, locally warm SSTs may contribute to the development and amplification of HHEs. During HHE peak period (D0 to D + 2), there is a positive SST anomaly of up to 0.2 °C across most of SEA (Fig. 5a), with stippling indicating regions where anomalies are significantly higher than non-HHE MAM days (*p* < 0.05).

To determine whether local SST anomalies act as a driver or a secondary response within ENSO and IOD coupling, a lead-lag correlation was calculated for each climate mode. Local SST anomalies were defined from 10 × 10 ocean grid cells surrounding each coastal HHE centroid. *T*_*w*_ anomalies at HHE onset (D0) were correlated with spatially averaged local SST anomalies across lags from −20 to +20 days (see Methods). The results indicate a lagged covariability between local SST anomalies and *T*_*w*_, with a higher correlation than that in the climatology, defined as the covariability across all MAM days regardless of HHE occurrence (Figs. 5b,c). To test significance, we constructed a null hypothesis via a bootstrap procedure (*N* = 1000), correlating *T*_*w*_ onset values with SST anomalies from randomly selected years at the same locations and seasonal timings as the observed HHEs.

During El Niño conditions (Fig. 5b), the maximum correlation window period ( + 0.40) is observed around 15–8 days before HHE onset (D-15–D-8) and is well above the mean climatological correlation ( + 0.16) and mean of the null hypothesis ( + 0.15), indicating that HHEs tend to occur after periods of increased SST anomalies. These pre-existing elevated SSTs likely enhance low-level atmospheric moisture availability, which supports HHE development. Similar mechanisms have been reported over mainland southern East Asia^43^, where warm SSTs contribute to the development and amplification of HHEs through moist air advection from nearby seas. Regression at this maximum correlation window period shows that a 1 °C increase in local SST is associated with an average increase of 0.34 °C (SE ± 0.02) in *T*_*w*_, with SST explaining 16% of *T*_*w*_ variance. Meanwhile, La Niña conditions exhibit a negative correlation (*r* = −0.23) between D-5 to D-2 (Fig. 5b), which suggests that HHE development is largely independent of local oceanic warming. Therefore, the increase in *T*_*w*_ in La Niña phases likely arises from atmospheric forcing and or land-atmosphere feedback mechanisms rather than by local SST anomalies.

Under positive IOD conditions (Fig. 5c), a weak relationship is present at D-15 to D-11 before HHE onset (*r* = +0.19). Although this exceeds the climatological mean (*r* = +0.16), it remains within the 95% confidence interval of the null distribution. During negative IOD conditions, a low correlation value at D-9 to D-5 (*r* = −0.11) also falls within this confidence interval. These results suggest that *T*_*w*_ increases during negative IOD events, like during La Niña, are not driven by local SST anomalies. Meanwhile, the correlation values under neutral condition predominantly appear after D0 and are statistically indistinguishable from the noise. In general, the relationships are, however, weak across all IOD phases, and if there is a true association between IOD-driven local SSTs, additional data is needed to detect it reliably.

### Role of ENSO and IOD

**Fig. 6:**
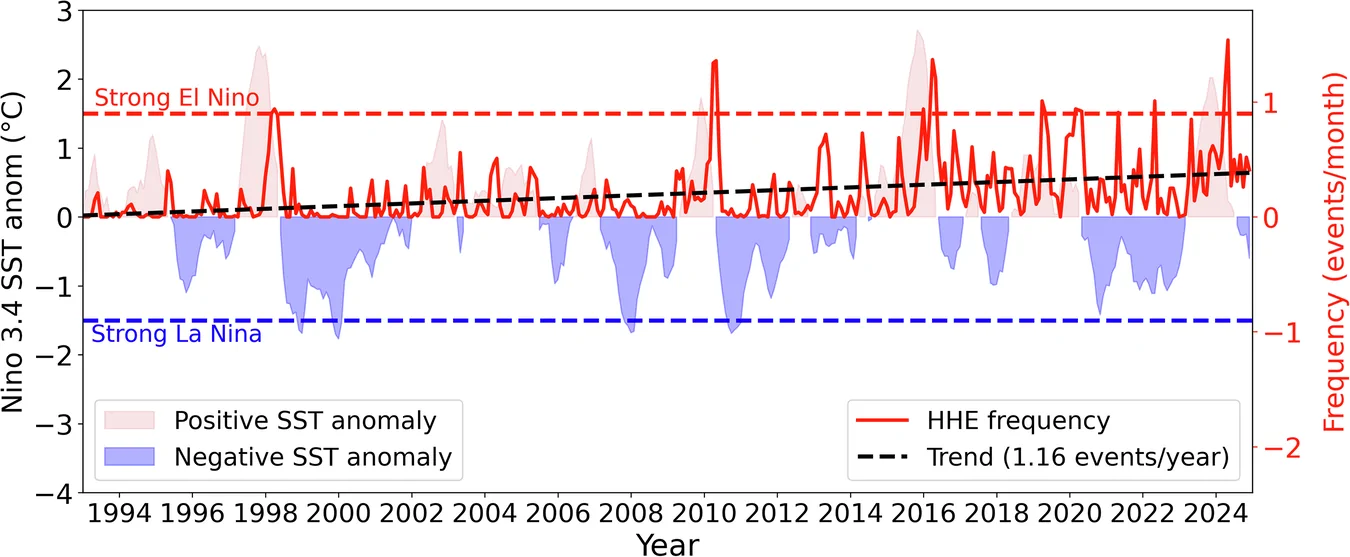
ENSO and equatorial SEA monthly HHE occurrence. Positive SST anomalies (°C; red shading, left axis) indicates El Niño conditions, meanwhile negative anomalies (°C; blue shading, left axis) indicate La Niña. The right y-axis shows the frequency of HHE events per month, as a mean over all land grids where at least one event occurs. Solid and dashed black lines denote long-term trends in event frequency. The trend is expressed as the fraction of affected land grids and events per year and is significant at the 5% level.

On interannual timescales, we examine the relationship between HHE variability and ENSO (Fig. 6). There were strong El Niño events in 1997–1998, 2009–2010, 2015-2016, and 2023-2024, which all peaked in the DJF season, and which coincided with peaks in HHE frequency in the following MAM. This seasonal offset suggests that ENSO exerts a delayed but substantial influence on the occurrence of HHEs across equatorial SEA, separate to the locally generated SST anomalies that lag the HHE events (Fig. 5). It is physically consistent with the mechanism of ENSO-induced free-tropospheric warming, where deep Pacific convection forces a delayed atmospheric adjustment over the SEA^47^. A lag correlation analysis reveals statistically significant correlations (*p* < 0.05) between the DJF Niño3.4 index and detrended MAM HHE frequency, with a correlation coefficient of approximately +0.73.

The IOD also modulates HHE variability (Fig. S5), primarily through mechanisms analogous to ENSO, however, the statistical link is slightly weaker and less consistent, i.e. positive IOD events in 1994 and 2006 were not followed by peaks in HHEs. There is a lagged correlation of +0.41 between the SON IOD index and detrended MAM HHE frequency, a lower degree of covariability than that observed in ENSO. The relatively weak signal likely reflects both the smaller frequency number of strong IOD events and their frequent co-occurrence with ENSO; for instance, the only two strong negative IOD events in the past 32 years both coincided with La Niña phases, making their independent effects difficult to isolate^48^.

Both El Niño and positive IOD phases create favourable conditions for HHEs through two primary physical mechanisms: oceanic and atmospheric circulation changes. First, as also discussed in the previous lead-lag analysis (Fig. 5b), the widespread positive SST anomalies associated with El Niño may contribute to warmer global background temperature and enhanced low-level moisture availability prior to the onset of HHEs. Second, these interannual modes drive changes in the atmospheric circulation^32,47^. During El Niño, Walker circulation weakens and shifts eastward^13,30,45^, inducing 500 hPa subsidence and anticyclonic anomalies across much of equatorial SEA (Fig. S5b). Positive IOD events result in broadly similar, albeit weaker in magnitude, descent pattern (Fig. S5e). These subsidence can suppress convection and increase incoming surface solar radiation, directly promoting the formation and persistence of heat events, similar to mechanisms well-documented over East Asia^13,49^. Furthermore, superimposed on this strong interannual variability is a statistically significant increase in the frequency of HHEs since 1993 (Fig. 6). The linear trend indicates an average increase of 1.16 HHE events per year relative to the 1993–2024 mean. This observed trend is consistent with previous studies on historical trend^3,4,6,9^ and future climate model projections^8^, which suggest that SEA will experience some of the most intense increases in HHEs by the late 21st century.

### Modulation by the Madden-Julian oscillation

**Fig. 7:**
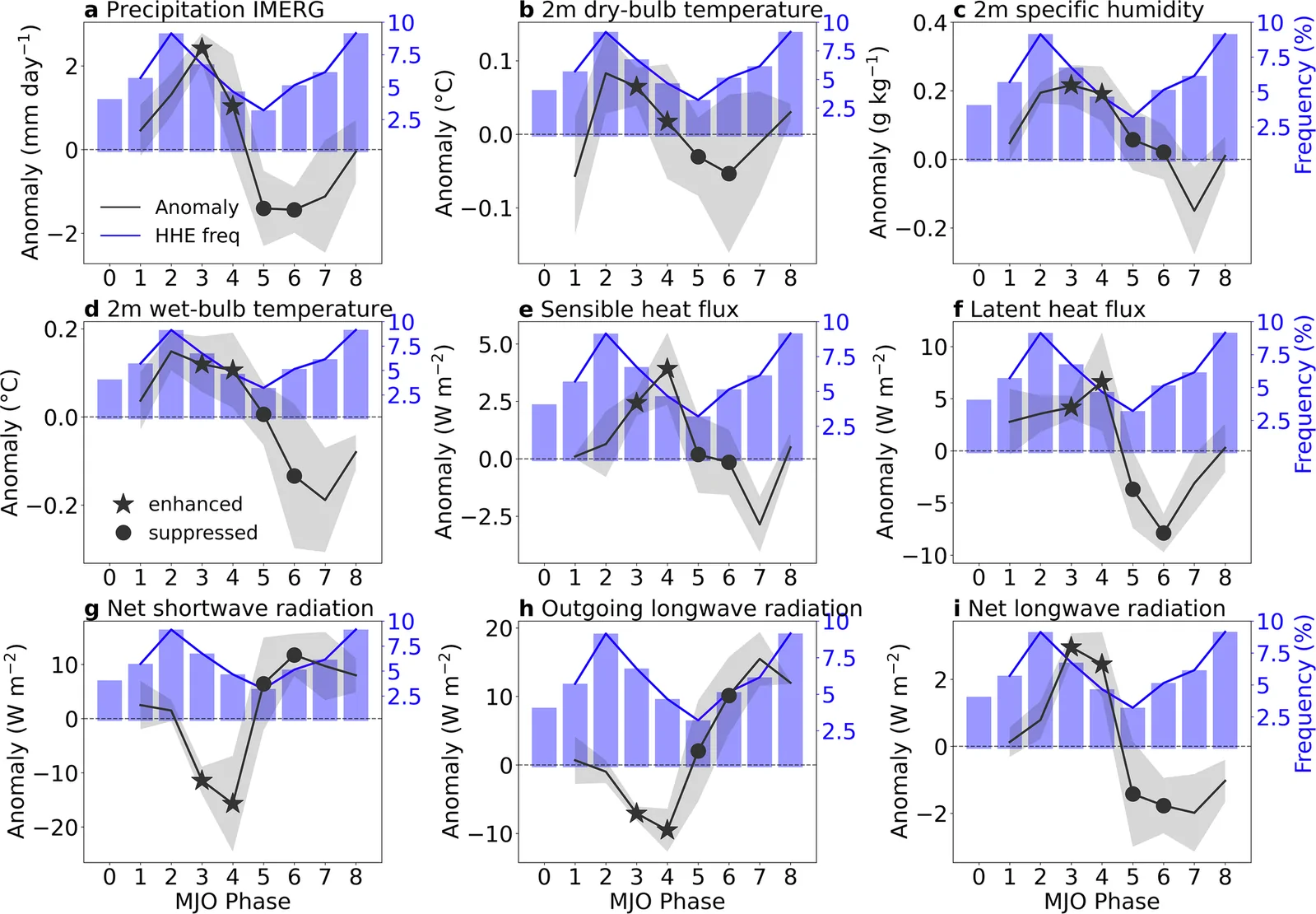
Anomalies of atmospheric variables in the West region by MJO phases during MAM period. Anomalies (black lines) of (**a**) precipitation IMERG, (**b**) 2m dry-bulb temperature, (**c**) 2m specific humidity, (**d**) 2m wet-bulb temperature, (**e**) sensible heat flux, (**f**) latent heat flux, (**g**) net shortwave radiation, (**h**) outgoing longwave radiation, and (**i**) net longwave radiation are computed over land grid cells only and are relative to the 15-day smoothed local daily climatology (1993 – 2024), except panel (**a**) which covers 2001–2024. Star symbols denote the enhanced MJO phases (3,4), circle symbols the suppressed MJO phases (5,6), while the neutral MJO phase (0) and transition phase (1,2,7,8) are shown without symbols. Grey shaded areas represent the 95% confidence intervals. Note that these composites represent all active MJO phases, not only those occurring during HHEs. The blue bars represent the relative frequency of HHE days per phase, expressed as a percentage.

To illustrate how the MJO modulates HHEs, we examine anomalies of key variables by MJO phase on all days in the MAM period (Fig. 7). The analysis focuses on the West region, but the modulations are found to be consistent across SEA, differing primarily in the timing of anomalies by phase as the MJO propagates eastward (not shown).

The transition to enhanced MJO phases begins in phase 2 and continues through phases 3–4 in the West region when convection intensifies^24,50–52^. During these phases, positive precipitation anomalies (1.0–2.4 mm h^-1^) occur over land (Fig. 7a). In phase 2, the large-scale active convective envelope resides over the Indian Ocean, immediately west of the equatorial SEA and coincides with generally positive anomalies in *T*_*2m*_, *q*_*2m*_, and *T*_*w*_ (Fig. 7b–d). This phase is characterised by intense moisture convergence in the boundary layer that precedes the peak of deep convection^50,52^ and aligns with one of the two peaks in HHE occurrence.

By phase 3, the arrival of the convective envelope produces peak rainfall anomalies (Fig. 7a). This shift leads to a subsequent decline in HHE variability. Consistent with recent work^40^, HHE frequency therefore peaks prior to the maximum precipitation. Once the MJO progresses to phase 4, precipitation anomalies begin to decline, yet the surface energy exchange intensifies. The peak in turbulent fluxes (Fig. 7e, f), particularly the latent heat flux, likely represents a response to the rainfall discharge in phase 3 and enhanced surface winds. Previous work shows that although moisture convergence begins to decrease during this phase, equivalent potential temperature remains positive near the surface, maintaining conditionally instability^50^. This, together with reduced downward solar radiation of up to 18 W m^−^^2^ in phase 4 (Fig. 7g), tends to suppress further near-surface warming and HHE frequency.

In contrast, the suppressed phases (5–6) are characterised by lower *q*_*2m*_ anomalies (Fig. 7c), driven by an increase in incoming shortwave radiation of approximately 10 W m^−^^2^ in phase 5 – 6 (Fig. 7g). Reduced cloud cover (Fig. 7h) drives increases of incoming shortwave radiation and small decreases in upwelling longwave radiation of around 2 W m^−^^2^ (Fig. 7i). This atmospheric drying arises from large-scale moisture deficits, with suppressed phases marked by strongly negative precipitation anomalies (Fig. 7a). These combined processes deplete atmospheric moisture, resulting in weak *T*_*w*_ anomalies (Fig. 7d) and a reduced likelihood of HHE occurrence. In phases 7, 8, and 1, near-surface humidity and temperature trend back toward climatological averages, with phase 8 exhibits a more pronounced positive anomaly in net shortwave radiation and reduced cloud cover. Moreover, a recent study found that the co-occurrence of phase 8 with El Niño may further reinforces the regional increase in temperature, as associated teleconnections tend to persist longer and sustain warming anomalies^53^, helping to explain the elevated HHE frequency in this phase.

Our results indicate a latitudinal shift in the MJO influence on SEA HHEs. While previous studies^11,29^ have linked MJO phase 4 to heat extremes over mainland SEA (e.g. Myanmar, Laos, Thailand, and Cambodia) through the westward expansion of the Western Pacific Subtropical High (WPSH), the peak HHE risk in most of the equatorial SEA occurs during MJO phases 2 and 8. These results suggest that equatorial HHEs are less directly governed by subtropical ridge dynamics.

### Modulation by equatorial waves

**Fig. 8:**
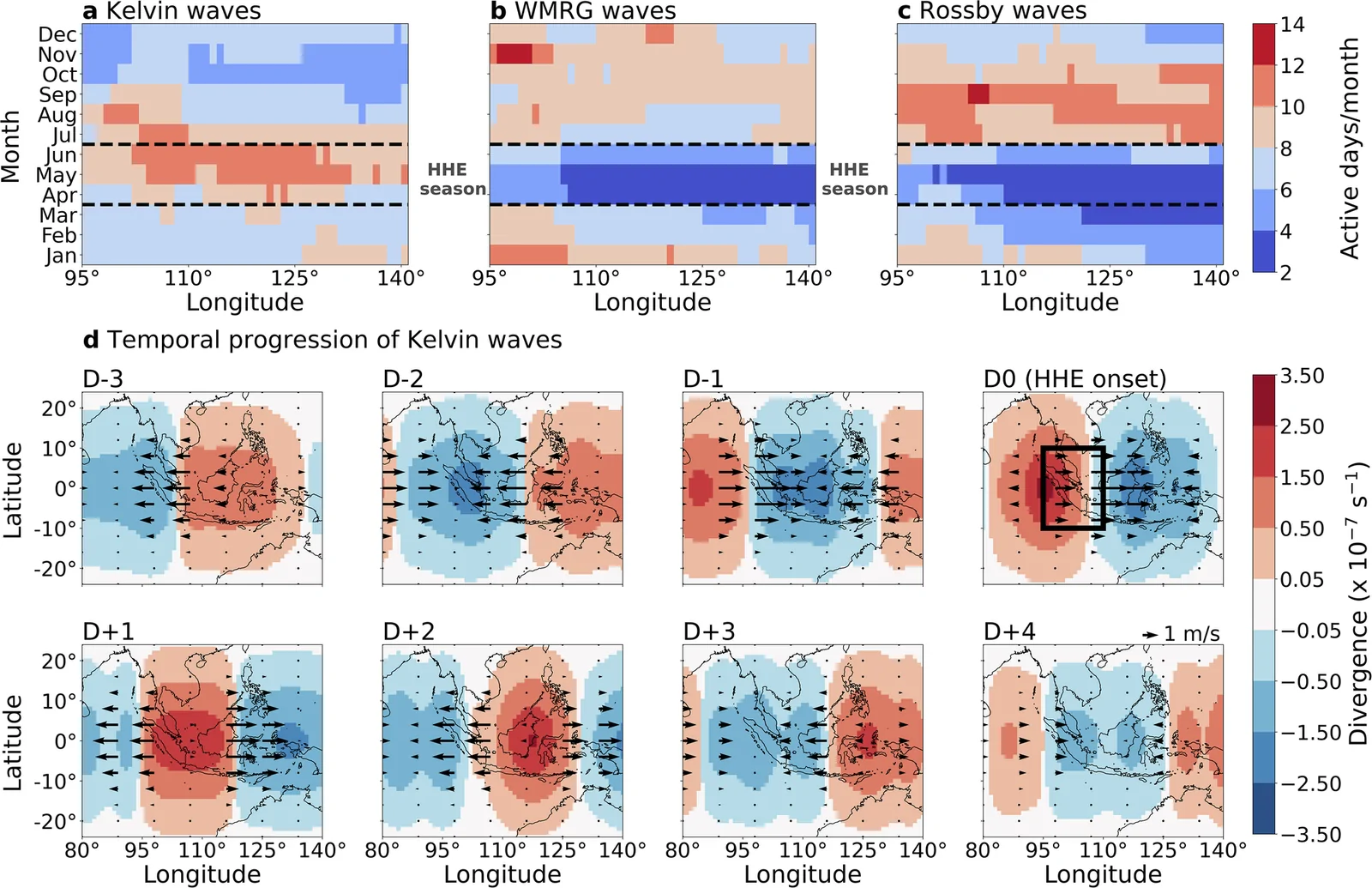
Climatology of equatorial wave modes across SEA and temporal progression of Kelvin waves during HHEs in the West region (95° –109°E). Hovmöller plots showing the climatological mean number of active days for Kelvin (**a**), MRG (**b**), and Rossby waves (**c**), averaged across 10°N – 10°S. The horizontal dashed lines indicate the peak HHE period (MAM). **d** 850 hPa divergence (s^-1^; shading) and zonal wind (ms^−1^) anomalies, from three days before (D-3) to four days after (D + 4) the HHE onset day (D0). The black rectangle in the D0 panel represents the West region. Anomalies are calculated relative to 1993–2024 daily means and smoothed using a 15-day moving average.

Equatorial Kelvin waves typically propagate eastward along the equator with periods of approximately 2.5–17 days^26,54,55^. Equatorial WMRG and Rossby waves travel westward and have periods ranging from ~3–6 days^52^ and 10–30 days^55,56^, respectively. These shorter periods, relative to those of the MJO, ENSO and IOD, make equatorial waves ideal candidates for influencing HHE events that have a typical length of 3 or 4 days. We use an equatorial waves dataset provided by Yang et al.^57^, where dynamical fields from ERA5 are projected onto theoretical wave structures to isolate the contribution of each mode at the 850 hPa level (see Methods). Equatorial Kelvin waves peak between March and July, when they are active 56% of the time (Fig. 8a), a period that aligns with the peak HHE season (MAM). In contrast, WMRG and Rossby waves are only active 39% and 37% of the time during the HHE season (Fig. 8b, c).

There is a clear transition through time when the structure of Kelvin waves is composited onto HHEs over the West region (Fig. 8d), a pattern that is consistent across equatorial SEA and separate results are therefore not shown. Approximately two days before HHE onset (D-2), low-level convergence and westerly wind anomalies associated with the convective phase of Kelvin waves pass over the West region. At HHE onset (D0), the pattern rapidly transitions to widespread low-level divergence and then anomalous easterly winds on D + 1. The subsequent return of the convergence phase around D + 3 align in time with the termination of favourable HHE conditions. The rapid lifecycle of Kelvin waves appears to drive HHE events but also confine the events to durations of three or four days (Fig. 1e). This suggests that Kelvin waves play a significant role in modulating daily variability of heat extreme as well as rainfall^23,58^. Their role in modulating heat extremes in the West region relative to the other intra and inter-seasonal tropical modes will be quantified in the following section, including a comparison with the East and Centre regions.

Unlike in mid- and high-latitudes where Rossby wave trains are the primary drivers of heatwaves^21,43^, HHEs over the equatorial SEA show the strongest association with Kelvin waves. The role of WMRG waves appears to be minimal, with WMRG waves driving only very weak variability in 850 hPa divergence and wind speed (Fig. S6a). While equatorial Rossby waves are associated with a low-level divergent pattern over continental SEA during HHE onset (Fig. S6b), they exert an opposing, convergent influence in equatorial SEA. This contrasting behaviour reflects the off-equatorial structure of Rossby waves, which limits their direct influence near the equator^57^.

**Fig. 9:**
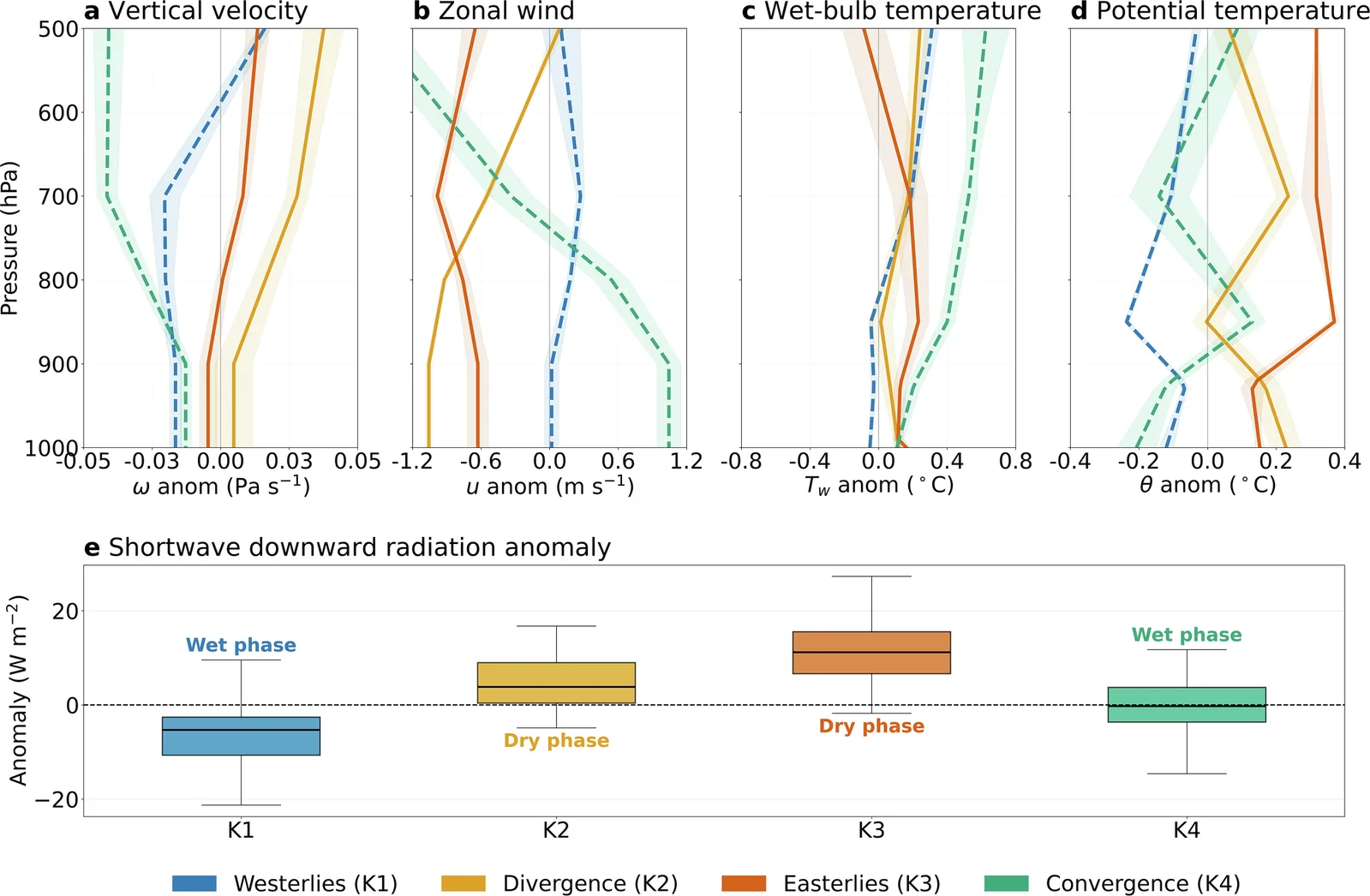
Composites of the vertical atmospheric structure during active equatorial Kelvin wave phases. **a** Vertical wind (*ω*, Pa s^-1^), **b** zonal wind (*u*, m s^-1^), **c** *T*_*w*_ (°C), **d** potential temperature (θ, °C) anomalies for wet (dashed line) and dry (solid line) Kelvin wave phases. **e** Shortwave downward radiation anomaly (W m^-2^) for each phase. The phases are labelled K1–K4 following the cycle defined by Crook et al.^23^: K1 (Westerlies), K2 (Divergence), K3 (Easterlies), and K4 (Convergence). Anomalies are for MAM relative to the 1993–2024 climatology, averaged over 10°S-10°N and 95°-109°E. Note that these composites represent all active Kelvin waves, not only those occurring during HHEs.

Kelvin wave influences on HHEs are now examined through variability in atmospheric vertical structure and shortwave heating during all active MAM Kelvin waves (i.e. not just during HHEs) across equatorial SEA (Fig. 9). The analysis focuses on the four canonical Kelvin wave phases^23,57^: westerlies and convergence (wet phases), and divergence and easterlies (dry phases). The dry phases of Kelvin waves are associated with subsidence at mid-levels (Fig. 9a), with upper-level convergence at 500 hPa present in the divergence phase (Fig. 9b). Easterly wind anomalies prevail throughout the vertical profile during both the divergence and easterly phases. There are positive *T*_*w*_ (Fig. 9c) and potential temperature (Fig. 9d) anomalies in the two dry phases, representing a warming response sustained by reduced cloud cover and a corresponding positive anomaly in downward shortwave radiation (Fig. 9e).

The peak in incoming solar radiation and atmospheric warming is observed during the easterly phase rather than the divergence phase. This phase offset likely reflects the behaviour of the wet Kelvin waves in the broader eastern hemisphere^59^, where convection aligns with the westerly phase rather than the centre of convergence phase. Consequently, the suppressed convection centre in this study is shifted west of the lower-tropospheric divergence maximum. The resulting column heating is much more intense in Kelvin waves than that produced by equatorial Rossby or WMRG wave (Fig. S7). Additionally, an increase of approximately 3 hPa in boundary layer height during the dry phases (not shown), likely represents surface heating response. Together, these features precondition the environment for the HHEs by coupling radiative surface warming with boundary layer deepening and convective suppression.

In contrast, the wet Kelvin wave phases are characterised by upward vertical velocity, westerly wind anomalies, and decreased potential temperature and downward shortwave radiation. Surface *T*_*w*_ anomalies remain minimal during these phases (Fig. 9c), however, enhanced low-level convergence promotes moisture accumulation aloft. This moisture buildup, in combination with unstable thermodynamic profiles, supports the initiation of deep convection. Nonetheless, such conditions are unfavourable for the onset of HHEs.

Whether modulated by the rapid lifecycle of equatorial Kelvin waves or the intraseasonal progression of the MJO, the transition from a typical warm day to a HHE day relies on a shared vertical atmospheric pathway. An extreme event occurs when surface intensification is accompanied by vertically coherent, favourable anomalies across the atmospheric column. Composite vertical profiles during MJO (Fig. S8) and equatorial Kelvin waves (Fig. S9) show that HHEs are characterised by an intensification of both potential temperature and specific humidity extending from the surface to 500 hPa. While zonal wind anomalies become stronger during HHE days, vertical velocity remains comparable to non-HHE days.

### Relative importance of tropical modes on HHE variability

**Fig. 10:**
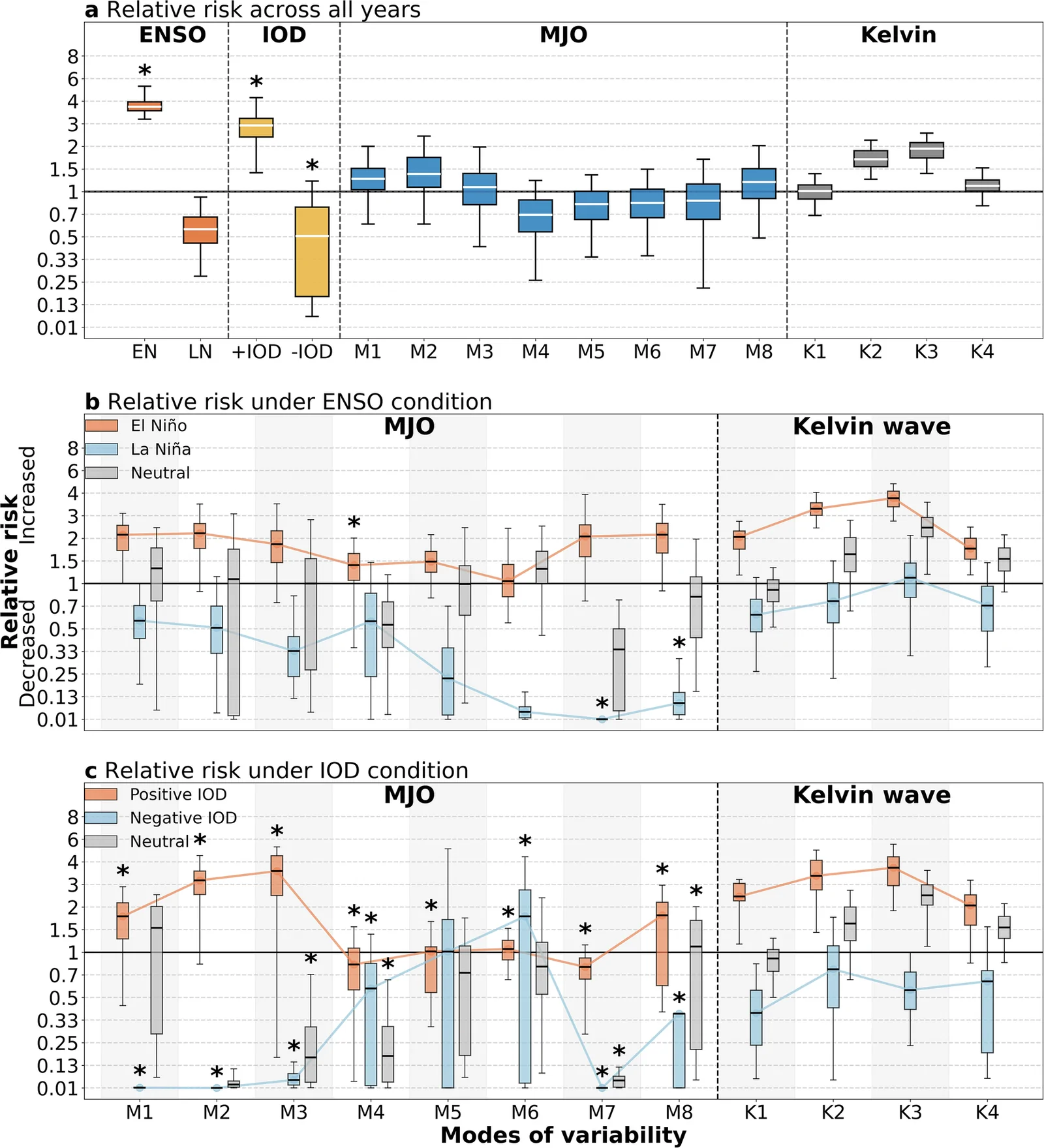
Relative risk of HHE occurrences associated with modes of variability in the West region during MAM. **a** Relative risk of HHE occurrence during the active phases of each mode relative to that mode’s neutral phase for the 1993-2024 period: ENSO (EN: El Niño, LN: La Niña), IOD ( + IOD: positive IOD, -IOD: negative IOD), MJO (M[number]: MJO in phase 1-8), and Kelvin waves (K1: westerlies, K2: divergence, K3: easterlies, K4: convergence). **b** Relative risk of MJO and Kelvin wave phases conditioned on ENSO state. **c** As in (b), but conditioned on IOD state. Orange (blue) lines connect the median relative risk of coupled MJO – Kelvin and El Niño - Positive IOD (La Niña – Negative IOD) phases. In all panels, the boxes show the interquartile range, the centre line represents the median, and the whiskers indicate the 2.5th and 97.5th percentiles of the bootstrap distribution. The asterisk (*) symbols above the upper whiskers represent a smaller sample size ( ≤ 10 active years for unconditioned interannual modes; ( ≤ 30 active days for intraseasonal and conditioned phases) for the corresponding composite. Relative risk values are calculated from the 32-year daily climatology from 1993–2024, with statistical uncertainty is assessed using 1000 bootstrap resamples within each active phase category.

We compute the relative risk (see Methods) of HHEs during each active phase of ENSO, IOD, MJO, and Kelvin waves relative to their neutral phases for the West region (Fig. 10) and the Centre and East regions (Figs. S10, 11), together with the associated event counts (Table S1). Taking El Niño as an example, the interpretation of relative risk is that a value greater than 1 indicates an increased risk of HHE occurrence in El Niño years compared to the risk of HHE occurrence in ENSO neutral years. A value of 2 indicates twice the risk and a value of 0.5 indicates that the risk is halved. Finally, a value of 1 (baseline) indicates no difference relative to the neutral phase.

Across all three regions, ENSO exhibits the most prominent association with HHE occurrence, with median relative risk values of 3–5 for El Niño (EN) and 0.5–0.6 for La Niña (LN), with most bootstrap confidence intervals separated from the baseline. This suggests a statistically strong and consistent association, aligned with previous studies which linked El Niño to elevated *T*_*w*_ over tropical land and to prolonged, slower-moving, and continuous heat extremes^14,47^. The IOD also shows a notable association with HHEs in all three regions, with median relative risk values of 3–5 in the positive phase ( + IOD) and 0.4–0.5 in the negative phase (-IOD). The IOD relative risk is calculated while controlling for the positive ENSO phase, ensuring that the reported associations reflect the independent contribution of the IOD. However, the broader spread of IOD relative risk indicates greater uncertainty compared to ENSO.

The West region demonstrates observable variations in MJO relative risk, with phases 1,2,8 pointing to potentially enhanced risks (median values up to 1.2–1.4) and phases 4–7 having suppressed risks down to 0.7–0.9. Across most of the regions, the median relative risk tends to be higher during phase 2 and 8, where the bootstrap medians remain positioned above the baseline. Although the 2.5th–97.5th percentile whiskers occasionally intersect the baseline, the general tendency in the median risk suggests that these phases provide a conditional enhancement of HHE risk. Results are broadly similar for the Centre (Fig. S10) but are noisier in the East (Fig. S11), possibly due to the lower sample size of HHE events there.

The modulation associated with equatorial Kelvin wave phase appears more pronounced, with median relative risk values of 1.7 and 2.0 for the divergence (K2) and easterly (K3) phases compared to when Kelvin wave amplitude is weak. This sensitivity remains evident in the Centre region, where median relative risk values range from 1.3–1.8, but is weaker in the East (1.0–1.3). Conversely, the westerly (K1) and convergent (K4) phases show relative risks close to the baseline ( ~ 1.0) which indicates a weak influence on HHE occurrence. Meanwhile, the WMRG and Rossby waves have only a weak influence on HHEs across all their phases (0.7–1.0, Fig. S12), which is consistent with their comparatively weak low-level divergence and associated column heating required to enhance temperature extremes in the equatorial SEA.

We now investigate whether the relative risk by MJO and Kelvin waves varies under different ENSO and the IOD conditions. When the West region relative risk by MJO and Kelvin phase is split into the three ENSO states (El Niño, La Niña, neutral), the shape of the variability remains reasonably similar, and it is the values of relative risk which change considerably (Fig. 10b). El Niño amplifies the relative risk values to above one across all MJO and Kelvin phases, whereas La Niña suppresses them to below one. When ENSO and IOD are neutral, the MJO phase signals appear noisy, whereas the Kelvin wave phases remain more consistent (Fig. 10b, c). Under the influence of Kelvin waves alone, HHE risk ranges from 1.6 in K2 to 2.5 in K3, slightly lower than during co-occurrence with El Niño, when the relative risk increases to 2 in K2 and 3 in K3. El Niño appears to provide low-frequency thermodynamic preconditioning that elevates baseline the temperature and humidity, allowing MJO and Kelvin waves to more effectively modulate HHE relative frequencies.

To disentangle the independent contribution of the overlapping tropical modes, we applied a multivariate negative binomial generalised linear model (GLM; see Supplementary Methods B and Fig. S13). While empirical relative risk (Fig. 10) captures total observed risk, the GLM isolates independent multiplicative effects. Considering the influence of individual drivers, the relative risks predicted by the model are generally consistent with the empirical relative risk results, with El Niño showing the largest multiplicative-scale effect, followed by +IOD, the combined equatorial Kelvin wave (K2,3), and MJO (M1,2,8) group phases.

### Comparison with dry heat extremes

**Fig. 11:**
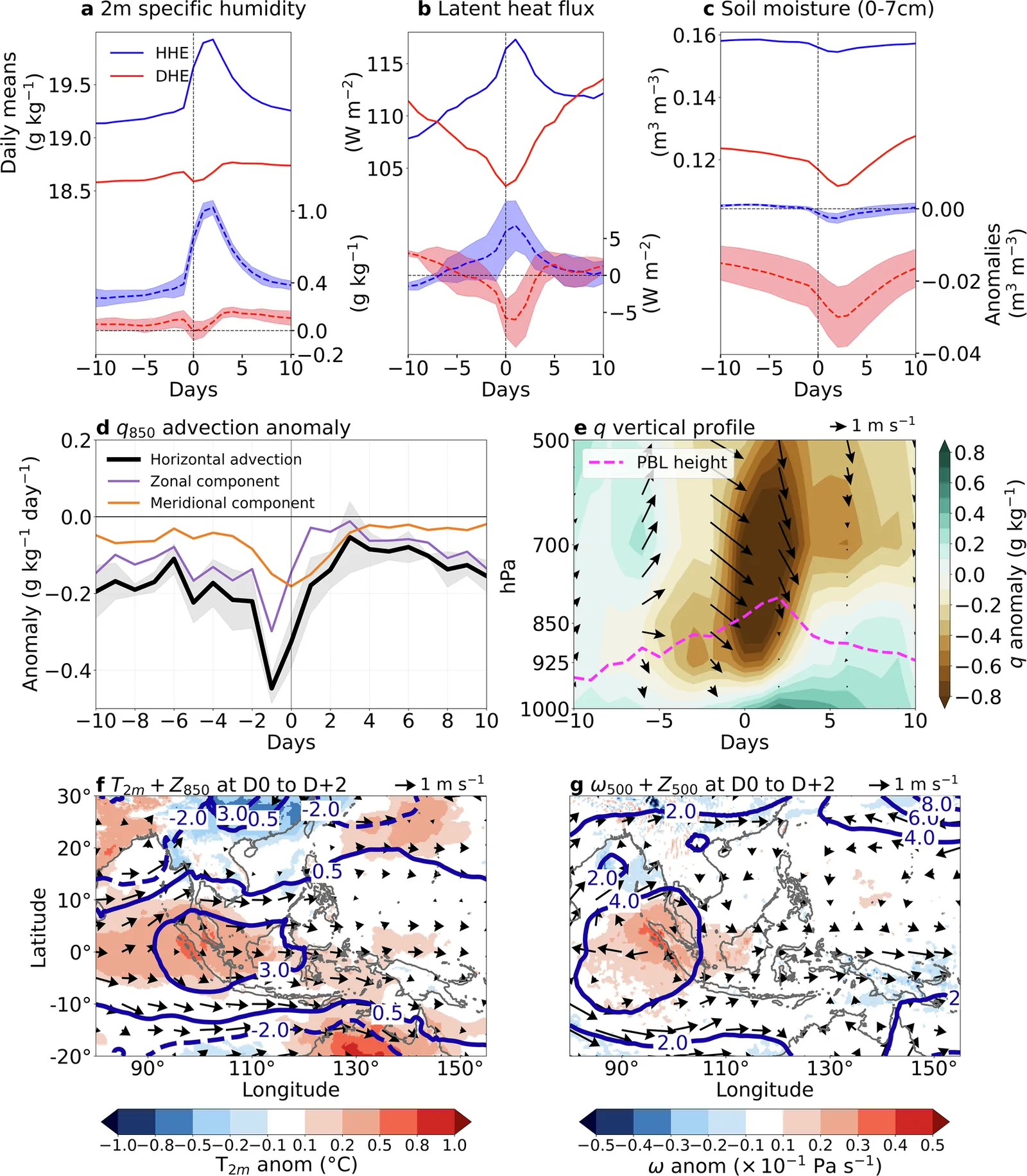
Key characteristics during DHEs in the West region. **a** Composite time series of *q*_*2m*_ daily mean values (solid lines) and anomalies (dashed lines). **b** As in (**a**), but for latent heat flux. **c** As in (**a**), but for surface soil moisture. **d** Composite time series of 850 hPa horizontal *q* advection anomaly (black line) and its corresponding decomposition into zonal (purple) and meridional (orange) components. Shaded areas denote 95% confidence intervals. **e** Time-pressure cross-section of anomalous *q* (g kg^-1^, shaded) and vertical-zonal wind vectors (m s^-1^, vector), with purple dashed-line indicates the daily maximum PBL height in hPa. **f** Anomalies of *T*_*2m*_ (°C; shading), with anomalies of 850 hPa geopotential height (*Z*, contours) and wind (*UV*, vector). **g** Anomalies of 500 hPa vertical velocity (*ω*, Pa s⁻¹; shading), geopotential height (m; contours), and wind (m s^-1^; vectors). Positive *ω* anomalies correspond to anomalous downward motion (subsidence), while negative anomalies indicate anomalous upward motion (ascent). Anomalies are calculated relative to local climatological daily means for the 1993–2024 period, smoothed with a 15-day moving average.

Here we consider the key differences between HHEs and DHEs for the West region only, where previous analysis in this paper shows the signals are the most robust. HHE and DHE events have broadly similar relative risks associated with large-scale drivers (Fig. 10 vs Fig. S14), with DHE risk higher in MJO phases 1 and 8 (1.4–1.5) than phase 2 (1.1), while HHE risk is lower in phases 1 and 8 (1.2–1.3) than in phase 2 (1.4). Most HHE drivers shown in Fig. 3 are like those for DHEs, albeit with different amplitude, and are thus not shown, with the main exceptions being *q*_*2m*_, latent heat flux, and soil moisture (Fig. 11a–c). HHEs are characterised by a rise in *q*_*2m*_, whereas DHEs develop under slightly drier conditions, with anomalies near zero at the onset (D0; Fig. 11a). This reduced moisture comes with a lower latent heat flux anomaly (-5.1 W m^-2^ compared with climatology, Fig. 11b), and a soil moisture anomaly decline of 0.03 m^3^ m^-3^ by D + 2 (Fig. 11c).

The vertical and horizontal moisture transport patterns further distinguish HHEs from DHEs. During DHEs, the total *q*_*850*_ horizontal advection anomaly reaches a minimum of −0.43 g kg^−^^1^ day^−^^1^ on D-1 (Fig. 11d), compared with a maximum of +0.13 g kg^−^^1^ day^−^^1^ at HHE onset (D0; Fig. 4d), indicating a dry air intrusion that precedes the *T*_*2m*_ peak. The vertical profile of *q* anomalies (Fig. 11e) shows a deep layer of moisture depletion extending from the surface up to at least 500 hPa on D0 for DHEs, although a positive *q* anomaly grows in the lowest levels from D + 1 to D + 5, which is similar but weaker than that for HHEs (Fig. 4e). This widespread drying through most of the troposphere, except the lowest level, leads to a higher PBL height (803 hPa compared to 817 hPa in HHEs), which could further suppress cloud formation and increases shortwave radiation reaching the surface. HHEs do not exhibit this deep drying but instead maintain a moist boundary layer that prevents effective heat dissipation through evaporation.

Both HHEs and DHEs are linked to anomalous high-pressure at 850 and 500 hPa during the first three days of the heat extremes (D0–D + 2). However, DHEs are closely linked to westerly wind anomalies (Fig. 11f, g), whereas HHEs typically feature easterly wind anomalies (Fig. 4b, g). Unlike HHEs, the high-pressure anomalies at 850 hPa during DHEs is more spatially confined over the West region, accompanied by a subsidence at 500 hPa. This large-scale subsidence, combined with surface desiccation, facilitates the development of DHEs. While HHEs occur alongside a moist and relatively shallow boundary layer, DHEs are characterised by the coincidence of both surface drying and large-scale atmospheric descent.

While HHEs and DHEs in equatorial SEA can arise from distinct drivers, their near-surface temperature *–* humidity characteristics and contribution to *T*_*w*_ remain broadly similar. This contrasts with regions outside the equatorial tropics, such as Southern China^60^, where *T*_*w*_ during DHEs is more humidity-controlled, whereas *T*_*w*_ during HHEs tends to vary more in step with temperature. Luo et al.^46^ noted distinct mechanisms between Southern China DHEs and HHEs, showing that the latter are associated with increased cloud cover and weaker surface radiative heating. In equatorial SEA, however, net shortwave radiation retains a persistent influence during both extremes, albeit with a greater amplitude during DHEs (not shown). Similar radiative characteristics are also evident in equatorial Africa, e.g. Gulf of Guinea and Central Africa^61^, however HHEs typically occur around one month later than DHEs. Taken together, these comparisons suggest limited distinction between HHEs and DHEs in equatorial SEA compared with other regions.

## Discussion

This study provides comprehensive understanding of the dynamic and thermodynamic drivers of equatorial SEA heat extremes, and quantifies tity. Results indicate that HHEs are generated in situ through a combination of diabatic and adiabatic heating, rather than relying on the horizontal advection of distant warm and/or moist air. The capacity for such local heating to reach extreme heat thresholds is constrained by the regional background conditions^14,29,47,62,63^, controlled by interannual modes such as ENSO and the IOD. They pre-condition the atmosphere by elevating baseline temperature and humidity, thereby allowing the MJO and equatorial Kelvin waves to effectively modulate HHE risk.

Across the West, Central and East regions of equatorial SEA, El Niño and positive IOD events significantly increase HHE risk up to 3–5 times. In contrast, La Niña and negative IOD reduce the HHE risk to about 0.5–0.6 and 0.4–0.5 times, respectively. On daily timescales, dry-to-wet transitions of the MJO and the dry Kelvin wave phases act as important dynamical triggers. When interannual background states coincide with intraseasonal modes, El Niño compounds the subsidence-driven warming, elevating the combined HHE risk to above 1 across all MJO and Kelvin wave phases. While the co-occurrence of positive IOD and Kelvin waves demonstrates a broadly similar compounding effect, the results for positive IOD and the MJO are noisier. The larger variance in HHE risk during IOD events, despite the proximity of the mode’s SST footprint to equatorial SEA, aligns with the high internal variability and decaying eastward signal-to-noise ratio documented in a broader circulation-focused study^64^. In the context of HHEs, these dynamical inconsistencies of the IOD manifest as a less coherent and more stochastic HHE risk compared to the more robust influence of ENSO

In the absence of interannual modes such as ENSO or IOD, equatorial Kelvin waves provide a more statistically consistent control on HHE variability relative to the MJO. We find that the divergence and easterly phases of these waves trigger HHE initiation by promoting surface radiative warming and adiabatic warming through subsidence. Unlike WMRG and Rossby waves which are fragmented by the complex regional geography^23^, equatorially trapped Kelvin waves retain dynamical coherence across the region. In addition, the MAM HHE season in equatorial SEA also aligns with the peak season of Kelvin wave amplitude, whereas WMRG and Rossby waves are at their minimum at this time of year. These reasons likely underpin the stronger and more robust influence of Kelvin waves on equatorial SEA HHE variability identified in this study.

While this study quantifies the relative contributions of several tropical modes to HHE variability in equatorial SEA, several aspects remain less explored. Although we were able to assess the relative contribution of co-occurring pairs of El Niño (and positive IOD) with MJO and Kelvin waves, the limited length of the timeseries prevented us from looking at all combinations of ENSO, IOD, MJO and Kelvin waves together. In addition, Kelvin waves are known to often precede the enhanced MJO phases^52^, and the role of this should be the subject of future research. Furthermore, while this study focuses on daily to interannual drivers, the variation of HHEs on sub-daily timescales and geographically dependent diurnal cycle of *T*_*w*_^65^ remain an important next step for improving local forecasts. Additionally, ongoing climate change may alter tropical mode variability, thus it is essential to assess how the dominant tropical modes identified here are projected to evolve under different climate change scenarios, alongside projections of HHE occurrences.

The most relevant metric for impact-based forecasting and early warning in equatorial SEA is still unclear^66^. The regional national meteorological services currently use various metrics for extreme heat prediction and warning, e.g., HI (PAGASA in the Philippines, MetMalaysia in Malaysia), *T*_*2m*_ (BMKG in Indonesia), and Wet-Bulb Globe Temperature (MSS in Singapore). Since there is low variability in specific humidity over equatorial SEA, the distinction between HHEs and DHEs is minimal, particularly compared with moisture-limited regions elsewhere in the tropics and subtropics, where HHEs and DHEs can be driven by completely different processes^42,62,67^ and often occur at different times of the year^8,62,67^. The choice of heat metric for early warning systems, is therefore, likely to be less critical in equatorial SEA than in more arid regions. Detailed heat-health studies, similar to a recent paper linking Japanese heat stroke-related ambulance transportations to different heat stress metrics^68^, are required to fully determine the most appropriate metric for equatorial SEA.

## Methods

### ERA5 reanalysis

Daily means were calculated from hourly data on the native spatial grid (0.25° × 0.25°) for the European Centre for Medium-Range Weather Forecasts (ECMWF) fifth generation reanalysis (ERA5)^34,69^ from 1993–2024.

### GPM-IMERG data

Daily accumulated precipitation data from the Integrated Multi-satellitE Retrievals for GPM (IMERG) product were used for the period 2001–2024. The GPM-IMERG dataset^70^, originally available at a 0.1° × 0.1° spatial resolution, was re-gridded onto the ERA5 grid.

### Sea surface temperature

Daily global SST data at a spatial resolution of 0.05°, 1993–2024, were used from the European Space Agency’s Climate Change Initiative (ESA CCI)^71^. To quantify local oceanic influence on coastal HHEs as presented in Fig. 5, a local SST signal was derived from a 10 × 10 grid cell domain centred on each coastal HHE centroid. Coastal HHE locations were identified using a land–sea mask threshold of 0.5, ensuring proximity to at least one adjacent ocean cell. To ensure that the SST signal used in the lead-lag correlations in Fig. 5 represents true oceanic conditions, we applied the ESA CCI land-sea mask. Local SST values were calculated by averaging all non-masked ocean pixels within each 10 × 10 grid cell.

### ENSO

Monthly Niño-3.4 SST anomalies from the National Oceanic and Atmospheric Administration Climate Prediction Centre (NOAA CPC) from 1993–2024^72^ were aligned with the daily-averaged ERA5 datasets by assigning the monthly value to each day within its respective month. Following the operational definition by NOAA, El Niño and La Niña episodes were identified when the Niño-3.4 SST anomalies exceeded ±0.5 °C for a minimum of five consecutive overlapping three-month periods. Additionally, strong ENSO episodes were further classified when anomalies exceeded ±1.5 °C for the same duration.

### IOD

Anomalies of the IOD (i.e. the Dipole Mode Index, DMI) were provided by NOAA^73^, based on monthly SST anomalies for the period 1993–2024. Positive and negative IOD events were identified when the three-month running mean of the DMI exceeded ±0.4 °C for at least three consecutive months. The monthly IOD values were aligned with the daily ERA5 data by assigning the monthly value to each day within its respective month.

### MJO

The Wheeler-Hendon index^74^, based on the Tropical Monitoring Outlooks from Bureau of Meteorology (BOM) were used to define the MJO for the period 1993–2024. Days when the amplitude index ≥ 1 are considered active periods.

### Equatorial waves

Equatorial wave activity was identified using an ERA5-based dataset provided by Yang et al.^57^, which classifies wave types by meridional mode number (*n*): Kelvin waves (*n* = −1), Westward-moving Mixed Rossby-Gravity (WMRG) waves (*n* = 0), and Equatorial Rossby-1 (ER) waves (*n* = 1). The dataset provides 6-hourly wind fields at a spatial resolution of 1° × 1° across 28 vertical pressure levels, covering the period from 1993 to 2024. For each wave type, the zonal (*u*) and meridional (*v*) wind components at 850 hPa were reconstructed to characterise wave dynamics. Wave amplitude and phase were computed following Crook et al.^23^, using diagnostic variables (*W*_*1*_ and *W*_*2*_; see Table 1) at latitudes representative of each wave mode. Amplitudes of *W*_*1*_ and *W*_*2*_ were normalised using their standard deviation over the study period and are considered active when their amplitude is ≥1. The equatorial wave data were converted to daily means and re-gridded to match the spatial resolution of ERA5.

**Table 1:** Diagnostic variables *W*_*1*_ and *W*_*2*_ used for equatorial wave mode, adapted from Crook et al.^23^, where *u* and *v* are zonal and meridional wind, and *x* is distance in metres

Wave type	W_1_	W_1_ latitude	W_2_	W_2_ latitude
Kelvin	*u*	0°N	$\frac{\partial u}{\partial x}$	0°N
WMRG	*-u*	10°S	*v*	0°N
Equatorial Rossby	*-u*	0°N	*v*	8°N

### Identification of HHE

HHEs were identified for land areas within 10°S and 10°N and 95°-140°E for the period 1993–2024. The identification procedure involved the following steps:

- Daily mean 2-metre *T*_*w*_ values were computed separately for each land grid cell using ERA5 data via the Davies-Jone methods^75^ and Python code from Raymond^76^.
- The 95th percentile of *T*_*w*_ parameters was calculated from all days 1993-2024. The 95th percentile is used to capture the most extreme heat events relative to the local climatology^39^, a stricter criterion than the 90th percentile commonly used in previous heat extremes studies^77,78^. Additionally, to exclude days unlikely to pose significant health risks, an absolute minimum threshold of 25.5°C was applied.
- Hot–humid days were identified when *T*_*w*_ exceeds both the local 95th percentile and the absolute minimum threshold of 25.5°C.
- A three-dimensional connected components algorithm^79^ (available via https://pypi.org/project/connected-components-3d/) was applied to aggregate the hot humid days into spatially contiguous HHEs.
- HHEs are defined as hot humid days occurring for a minimum duration of 3 days, and over a minimum spatial extent of 3 grid cells ( ~ 2,300 km^2^).
- DHEs were identified in the same way as above but using daily mean *T*_*2m*_ with a minimum absolute threshold of 28.5 °C.
- The climatology for HHEs, DHEs, and associated meteorological variables were smoothed using a 15-day window and averaged over the 32-year reference period (1993–2024).

*T*_*w*_ was selected to represent HHEs because it relates closely to surface thermodynamic properties of equivalent potential temperature (${\theta }_{e}$) and it is more strongly controlled by humidity than apparent temperature^80^ (*AT*) or wet-bulb globe temperature (*WBGT*)^81^, allowing for a more distinct categorisation of HHE and DHE events. *T*_*w*_ is also physiologically relevant as it captures the upper limit of evaporative cooling for healthy, well-acclimatised individuals under heat stress^8^. Physiological modelling by Vanos et al.^82^ suggest that adverse health effects may occur at *T*_*w*_ values^83^ between 22° and 34 °C^82^, much lower than the commonly cited upper limit of survivability of *T*_*w*_ = 35 °C^27^. Thus, a threshold of 25.5 °C was applied to define HHEs, ensuring both physiological relevance and a sufficient sample size for robust statistical analysis. However, *T*_*w*_ may overlook critical aspects of heat-related risk, particularly in arid or high-elevation regions where extreme humidity is uncommon. *T*_*w*_ also does not incorporate solar radiation and wind dynamics, that can influence human thermal perception and heat stress response. A threshold of 28.5 °C was selected for DHEs to ensure a balanced comparison between datasets, as this specific threshold yields a sample size comparable to that of HHEs.

The use of daily mean *T*_*w*_ and *T*_*2m*_ values allows for the representation of both daytime heating and nighttime cooling, capturing the full diurnal cycle and better reflects prolonged exposure to heat stress conditions than peak daily values. This approach is particularly relevant for HHEs over SEA, where nighttime temperatures have been rising at a faster rate than daytime temperatures^6^. Moreover, daily means account for the cumulative thermal burden across a 24 h period, which is more representative of sustained physiological stress than short-term daytime peaks alone. Previous analysis has shown that using daily maximum temperatures does not substantially alter the identification of HHEs^39^.

### Calculation of the HI

HI was calculated based on the multiple regression formulation developed by Rothfusz and published by the NOAA/National Weather Service^38^. This formula estimates the perceived temperature by combining air temperature and relative humidity. Adjustments to the original equation are applied under conditions of unusually low or high relative humidity, as well as when Heat Index values fall below 80 °F (26.7 °C), to improve accuracy across a broader range of environmental conditions^84^. While HI is a commonly used indicator of thermal discomfort, its empirical formulation based on Steadman’s physiological data^80,85^ limits its reliability under extreme temperature and humidity conditions^86,87^. Moreover, HI does not account for environmental factors^88^ such as solar radiation, wind speed, outdoor activity, or clothing.

### Validation of ERA5 with weather station observations

Observed and ERA5 daily mean *T*_*2m*_ and *T*_*w*_ during HHEs were composited in Fig. S15 to ensure ERA5 data quality and reliability. Observations are from 133 in-situ surface weather stations from the HadISD^89^ dataset. Stations were selected based on data completeness, including only those with at least one observation available for each three-hourly interval throughout the analysis period (Figs. S15a, b). Each weather station was paired with the overlapping ERA5 grid box and lapse-rate corrections were applied to the ERA5 reanalysis temperatures, using both dry-adiabatic and saturated-adiabatic lapse rates (Figs. S15c, d), to adjust for elevation differences between ERA5 grid cells and the in-situ observation sites.

The two datasets show broad agreement, especially in the timing of HHE onset, indicating that the events identified in ERA5 were also observed. ERA5, however, has a cold bias, even after applying the lapse-rate correction, which acts to largely reduce but not eliminate the discrepancy. This bias likely arises from several factors, including the spatial averaging inherent to ERA5 grid cells compared with point-based station measurements, unresolved local topography and elevation difference, and the influence of land use cover on station records.

### Calculation of relative risk

The relative risk, sometimes referred to as the risk ratio, was calculated using:

1 $${Relative}\,{risk}=\,\frac{P\left(x\,\right|\,a)}{P\left(x\,\right|\,n)}$$

where *x* represents the number of HHE events at each grid, *a* and *n* denote calendar days classified as the active and neutral phases, respectively. Thus, *P(x∣a)* and *P(x/n)* represent the probability of observing HHEs given the active or neutral phase. For MJO and Kelvin waves and all compound climate states (Fig. 10b, c), the baseline (*P(x/n)*) is strictly defined as the calendar days of the inactive intraseasonal state (Phase 0) occurring under a neutral background climate. Equation (1) was applied to each grid cell prior to regional aggregation and for each large-scale driver assessed in this study to quantify whether these drivers enhanced or suppressed the occurrence of heat extremes. Bootstrapping with 1000 resamples was used to generate confidence intervals. Modulation by each driver was considered significant at the 5% level where the 2.5th–97.5th percentile range of the bootstrapped distribution lies above the baseline value of 1.

### Composite analysis

Lead-lag composite analyses were performed by centring a temporal window on the first day of each HHE and DHE (D0) event. To create a composite time series, daily anomalies for each variable were extracted across a uniform period to capture the evolution from the pre-event (before D0), peak (D0–D + 2) and post-event (D + 3 onwards). Anomalies were calculated using a 15-day smoothed mean, selected after doing a sensitivity tests with 7-, 10-, and 20-day windows (not shown).

## Supplementary information

Hidayat_supplementary_materials

## Data Availability

The ERA5 reanalysis data are available from the Copernicus Climate Change Service https://doi.org/10.24381/cds.adbb2d47 for single level data^34^ and https://doi.org/10.1002/qj.4174 for pressure level data^69^. GPM-IMERG data are available from https://gpm1.gesdisc.eosdis.nasa.gov/data/GPM_L3/GPM_3IMERGDF.07/^70^. ENSO data are available from https://www.cpc.ncep.noaa.gov/products/analysis_monitoring/ensostuff/detrend.nino34.ascii.txt^72^. IOD data are available from https://psl.noaa.gov/gcos_wgsp/Timeseries/Data/dmi.had.long.data^73^. MJO data are available from http://www.bom.gov.au/climate/mjo/. The equatorial waves dataset is available on request from Yang et al.^57^ SST data are available from https://catalogue.ceda.ac.uk/uuid/4a9654136a7148e39b7feb56f8bb02d2/^71^. HadISD sub-daily station data^89^ are available from https://www.metoffice.gov.uk/hadobs/hadisd/v340_2023f/index.html.
