# Supplementary material for: Synoptic-to-interannual drivers of humid heat variability in equatorial Southeast Asia
Source: NPJ Clim Atmos Sci. 2026 Jun 9;9(1):221. doi: 10.1038/s41612-026-01451-3 (PMC13736710; doi:10.1038/s41612-026-01451-3)
Supplement: Supplementary file 1 [file 41612_2026_1451_MOESM1_ESM.pdf]

Supplementary Information

Synoptic-to-interannual drivers of humid heat variability in equatorial Southeast Asia

Anistia M. Hidayat[1,2*], Cathryn E. Birch[1], Lawrence S. Jackson[1], Juliane Schwendike[1], Claudio Sanchez[3], Adrian J. Matthews[4]

[1] School of Earth and Environment, University of Leeds, Leeds, LS2 9JT, UK.
[2] Indonesian Agency for Meteorology, Climatology, and Geophysics (BMKG), Jakarta, 10610, Indonesia.
[3] Met Office, Exeter, EX1 3PB, UK.
[4] School of Environmental Sciences, University of East Anglia, Norwich, NR4 7TJ, UK.

Correspondence to: Anistia M. Hidayat (eeamh@leeds.ac.uk)

Supplementary Methods

A. Lagrangian temperature budget analysis

To quantify the physical processes driving the near-surface temperature anomalies (*T′*) during HHEs, we applied the Lagrangian temperature-anomaly budget diagnostic developed by Röthlisberger and Papritz (2023). This framework utilises the thermodynamic energy equation in pressure coordinates to decompose temperature changes along air-parcel backward trajectories.

To ensure that the budget analysis remains focused on the triggering mechanisms near the ground, trajectories were initialised over each HHE locations at 10, 30, and 50 hPa levels above the surface. For a baseline surface pressure of 1,000 hPa, this corresponds to the 990, 970, and 950 hPa levels. From these near-surface arrival destinations, the air parcels were tracked backwards in time for 12 days. As the parcels were tracked backwards, they were allowed to move freely up and down through the lower troposphere based on the vertical wind velocity (*ω*). The physical budget along each trajectory was integrated using 6-hourly three-dimensional wind (*u, v, ω*) and temperature fields extracted from 12 ERA5 vertical levels, ranging from the surface up to 500 hPa.

By defining the temperature anomaly (*T′*) as the difference between the absolute temperature (*T*) and the transient climatology ($\overline{T}$), $T' = T - \overline{T}$, and expanding the material derivative of the climatological temperature, the rate of change of the temperature anomaly over time *t* along an air-parcel trajectory is expressed as:

$$\frac{DT'}{Dt} = -\frac{\partial \overline{T}}{\partial t} - \boldsymbol{v_h} \cdot \boldsymbol{\nabla_h} \overline{T} + \left[\frac{\kappa T}{p} - \frac{\partial \overline{T}}{\partial p}\right] \omega + \left(\frac{p}{p_0}\right)^{\kappa} \frac{D\theta}{Dt}, \tag{1}$$

where $\boldsymbol{v}_h$ is the horizontal wind vector, ω is the vertical velocity, θ is the potential temperature, κ is the Poisson constant (0.286), *p* is the transient pressure of the air parcels at any given point along their 3D trajectories, and p_0 is the reference surface pressure.

To decompose the final temperature anomaly at the event location and time, the terms on the right-hand side are integrated forward in time along the backward trajectory, starting from the anomaly genesis time. This yields four distinct physical components on the right-hand side of Eq. (1):

- **Seasonality** $\boldsymbol{T'}\left(-\frac{\partial \overline{T}}{\partial t}\right)$: the anomaly arising from changes in the background temperature climatology over the trajectory duration.
- **Advective** $\boldsymbol{T'}\left(-\boldsymbol{v_h} \cdot \boldsymbol{\nabla_h} \overline{T}\right)$: the anomaly generated by the horizontal advection of the air parcel across climatological temperature gradients.
- **Adiabatic** $\boldsymbol{T'}\left(\left[\frac{\kappa T}{p} - \frac{\partial \overline{T}}{\partial p}\right] \omega\right)$: the anomaly generated through vertical motion.
- **Diabatic** $\boldsymbol{T'}\left(\left(\frac{p}{p_0}\right)^{\kappa} \frac{D\theta}{Dt}\right)$: the anomaly caused by diabatic processes along the trajectory, such as surface sensible heat fluxes, turbulence, and radiation.

Limitation of Lagrangian temperature budget analysis for HHE study:

1. Under the assumption of a dry atmosphere, the diagnostic equation evaluates changes in temperature anomalies (*T′*) using only dry thermodynamic processes. Since the equation tracks sensible heating and adiabatic processes exclusively through dry air temperature, it does not explicitly represent moisture related processes. Consequently, while HHEs are physically defined by the co-occurrence of extreme heat and high humidity, this framework primarily captures the thermodynamic drivers of the sensible heat component, while the moisture evolution of the air parcel remains largely unresolved. As a result, processes such as surface latent heat fluxes (evapotranspiration) and low-level moisture advection cannot be directly quantified.

2. The framework demonstrates that temperature anomalies contributing to heat extremes over land can originally form over the ocean. In a warming climate, near-surface land–ocean temperature contrasts are projected to widen (Sutton et al., 2007; Byrne and O'Gorman, 2018). For a given onshore-moving trajectory, this widening contrast causes the advective *T'* to decrease more rapidly as it crosses the coastline, potentially weakening the resulting heat anomaly over coastal land.
3. Tracking air parcels near surface levels using 6-hourly resolved ERA5 gridded wind fields introduces inherent spatial and temporal limitations. The gridded dataset cannot fully resolve the sub-grid scale turbulent mixing, localised coastal sea breezes, and convective diurnal cycles characteristic of the tropical planetary boundary layer. Consequently, the parameterized turbulence near the ground introduces higher localized uncertainty regarding the exact historical path of the air parcels compared to trajectories tracked in the stable, advection-dominated free troposphere.
4. Tracking near-surface air parcels using 6-hourly ERA5 wind data has several limitations. The coarse spatial and temporal resolution cannot fully capture small-scale processes (Guo et al., 2021) in the tropical boundary layer, such as turbulent mixing, coastal sea breezes, and diurnal convection. As a result, air-parcel trajectories near the surface are subject to greater uncertainty, particularly regarding their exact past pathways, compared to trajectories in the more stable free troposphere where large-scale advection dominates.

References:

Guo, J. *et al.* Investigation of near-global daytime boundary layer height using high-resolution radiosondes: first results and comparison with ERA5, MERRA-2, JRA-55, and NCEP-2 reanalyses. *Atmos. Chem. Phys.* **21**, 17079–17097 (2021).

Sutton, R. T., Dong, B. & Gregory, J. M. Land/sea warming ratio in response to climate change: IPCC AR4 model results and comparison with observations. *Geophys. Res. Lett.* **34**, L02701 (2007).

Byrne, M. P. & O'Gorman, P. A. Trends in continental temperature and humidity directly linked to ocean warming. *Proc. Natl Acad. Sci. USA* **115**, 4863–4868 (2018).

B. Negative binomial generalised linear model (GLM)

To quantify the independent contributions and interaction of several tropical modes, we applied a multivariate Negative Binomial Generalised Linear Model (GLM) with log-link function. The model predicts the expected rate of HHEs (μ). To account for day-to-day variations in spatial data availability, the logarithm of the number of valid grid cells was included as an offset term. To reduce overfitting and unstable coefficients caused by sparse combination of tropical modes, the MJO and equatorial Kelvin wave phases were grouped into broader categorial variables (e.g. $M_{1,2,8,}$ $M_{4,5,6}$ $K_{2,3}$ and $K_{1,4}$). The full interaction model, isolating the multiplicative change in expected HHE occurrences relative to the neural background state, is expressed as:

$$\begin{aligned}\log(\mu) = {} & -142.1 + 1.3(\mathrm{EN}) - 0.87(\mathrm{LN}) + 0.16(+\mathrm{IOD}) - 0.43(-\mathrm{IOD}) \\ & -0.35(M_{4,5,6}) - 0.26(M_{1,2,8}) + 0.02(K_{1,4}) + 0.11(K_{2,3}) \\ & -3.2(\mathrm{LN})(-\mathrm{IOD}) + 2.3\times10^{15}(\mathrm{EN})(-\mathrm{IOD}) + 1.9\times10^{15}(\mathrm{LN})(+\mathrm{IOD}) \\ & +0.64(\mathrm{EN})(+\mathrm{IOD}) + 0.41(\mathrm{EN})(K_{1,4}) + 0.08(\mathrm{LN})(K_{2,3}) + 0.29(\mathrm{EN})(K_{2,3}) \\ & +0.08(\mathrm{LN})(M_{1,2,8}) + 0.50(\mathrm{EN})(M_{1,2,8}) - 0.35(\mathrm{LN})(M_{4,5,6}) + 0.11(\mathrm{EN})(M_{4,5,6}) \\ & -0.37(-\mathrm{IOD})(K_{1,4}) + 0.08(+\mathrm{IOD})(K_{1,4}) - 0.46(-\mathrm{IOD})(K_{2,3}) + 0.05(+\mathrm{IOD})(K_{2,3}) \\ & -1.91(-\mathrm{IOD})(M_{1,2,8}) + 0.02(+\mathrm{IOD})(M_{1,2,8}) + 0.04(-\mathrm{IOD})(M_{4,5,6}) \\ & +0.02(+\mathrm{IOD})(M_{4,5,6}) + 0.37(K_{1,4})(M_{1,2,8}) + 0.21(K_{2,3})(M_{1,2,8}) \\ & +0.004(K_{1,4})(M_{4,5,6}) - 0.16(K_{2,3})(M_{4,5,6}) + 0.07(\alpha),\end{aligned} \tag{2}$$

where model coefficients were exponentiated to obtain μ. The categorical predictors (EN, LN, +IOD, -IOD, $M_{4,5,6,}$ $M_{1,2,8}$, $K_{1,4}$, and $K_{2,3}$) correspond to the tropical modes defined in Fig. 10 and act as indicator function to Eq. (2), which evaluate to 1 if the categorical predictors occur and 0 if they are absent. Meanwhile, α represents the continuous variable year, included to account for the long-term warming trend.

To quantify the effects of these modes, we first fitted a baseline main-effects negative binomial model, which allowed us to derive the predicted μ for the individual, isolated modes. Next, to account for compound events, we fitted a secondary model that incorporated all pairwise interactions between the categorical tropical modes. We then compared these two models using a likelihood-ratio test to confirm that adding the interaction terms improved the model's predictive performance ($p < 0.001$). Finally, using the predictions from this validated interaction model (Eq. 2), we quantified the risk associated with co-occurring tropical climate modes by calculating the Interaction Ratio (IR) for a given pair of modes A and B as follows:

$$\mathrm{IR} = \frac{\mu_{11} \cdot \mu_{00}}{\mu_{10} \cdot \mu_{01}} \tag{3}$$

where:

- μ_{00}: mean HHE count when both are in the reference state
- μ_{10}: mean HHE count when A is active, B in reference
- μ_{01}: mean HHE count when B is active, A in reference
- μ_{11}: mean HHE count when both are active

Limitations of the GLM approach:

1. Complete separation and data sparsity: the inclusion of a full two-way interaction matrix forces the model to evaluate physically contradictory or exceptionally rare climate states (e.g., El Niño concurring with -IOD). The lack of empirical data in these intersections causes the log-link function to produce infinitely large, standard errors, causing those specific interaction terms physically meaningless.
2. The multiplicative constraint vs. thermodynamic limits: the GLM relies on a log-link function, which assumes that risk scales multiplicatively without bound. However, the atmosphere is constrained by physical thermodynamic ceilings (e.g., the Clausius-Clapeyron relation). The GLM cannot model these physical caps where the atmosphere stops heating and instead triggers precipitation.
3. Ignorance of temporal autocorrelation: HHEs are persistent events, if a grid cell experiences an HHE on Tuesday, the probability of an HHE on Wednesday is inherently elevated. The standard GLM treats every daily observation as statistically independent, which potentially violates the assumption of independence (i.i.d.) and artificially inflates the model's degrees of freedom and can result in overconfident (too small) p-values.

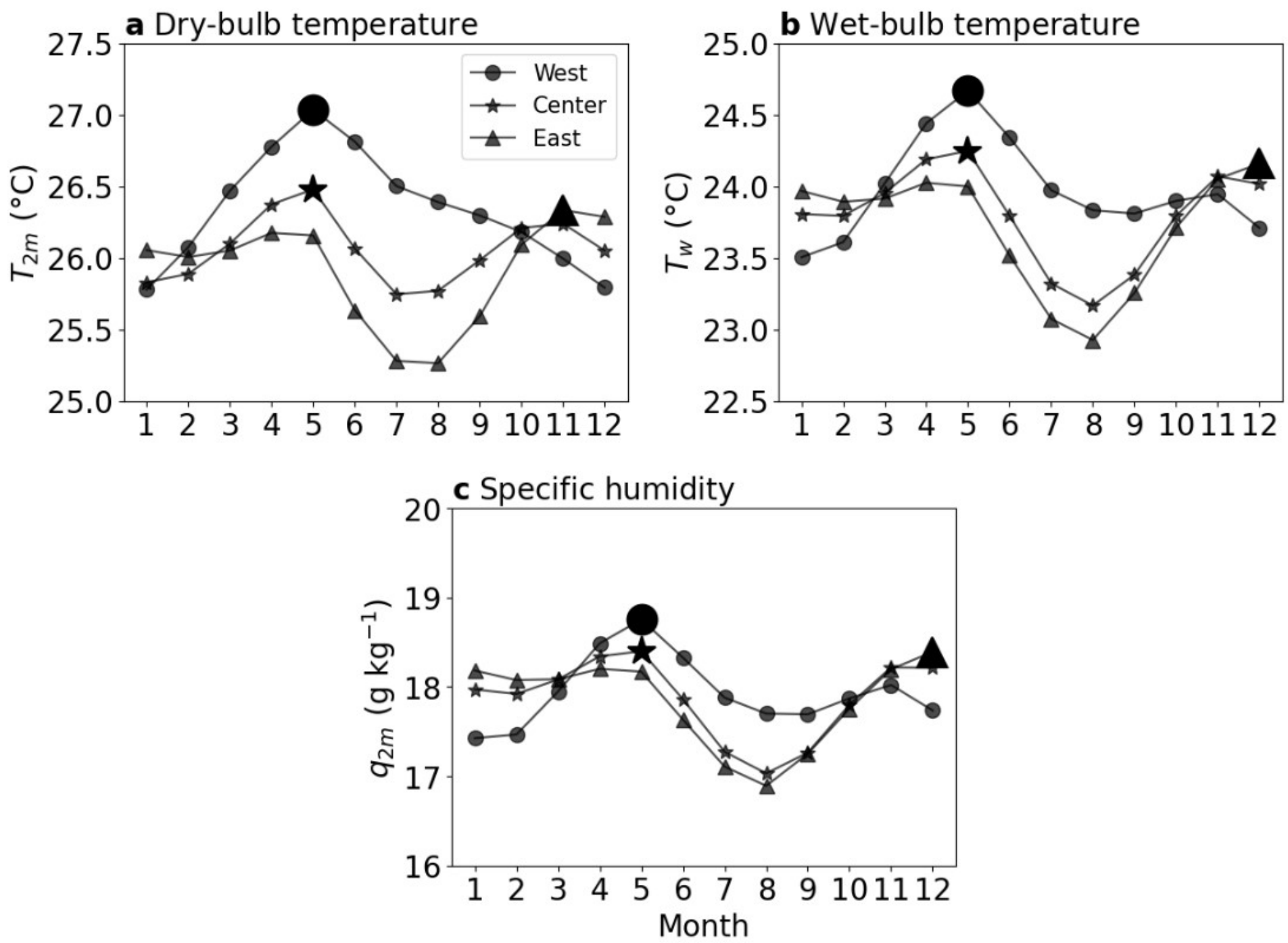

Supplementary Figure 1. Mean annual cycle of near-surface variables. a 2m dry-bulb temperature (T_{2m}, °C), **b** 2m wet-bulb temperature (T_w, °C), and **c** 2m specific humidity (q_{2m}, g kg^{-1}) are derived using ERA5 reanalysis data from 1993 to 2024 across the West (circles), Centre (stars), and East (triangles) regions. The larger symbol on each region indicates the month when the value of each variable reaches its maximum compared to other months of the year.

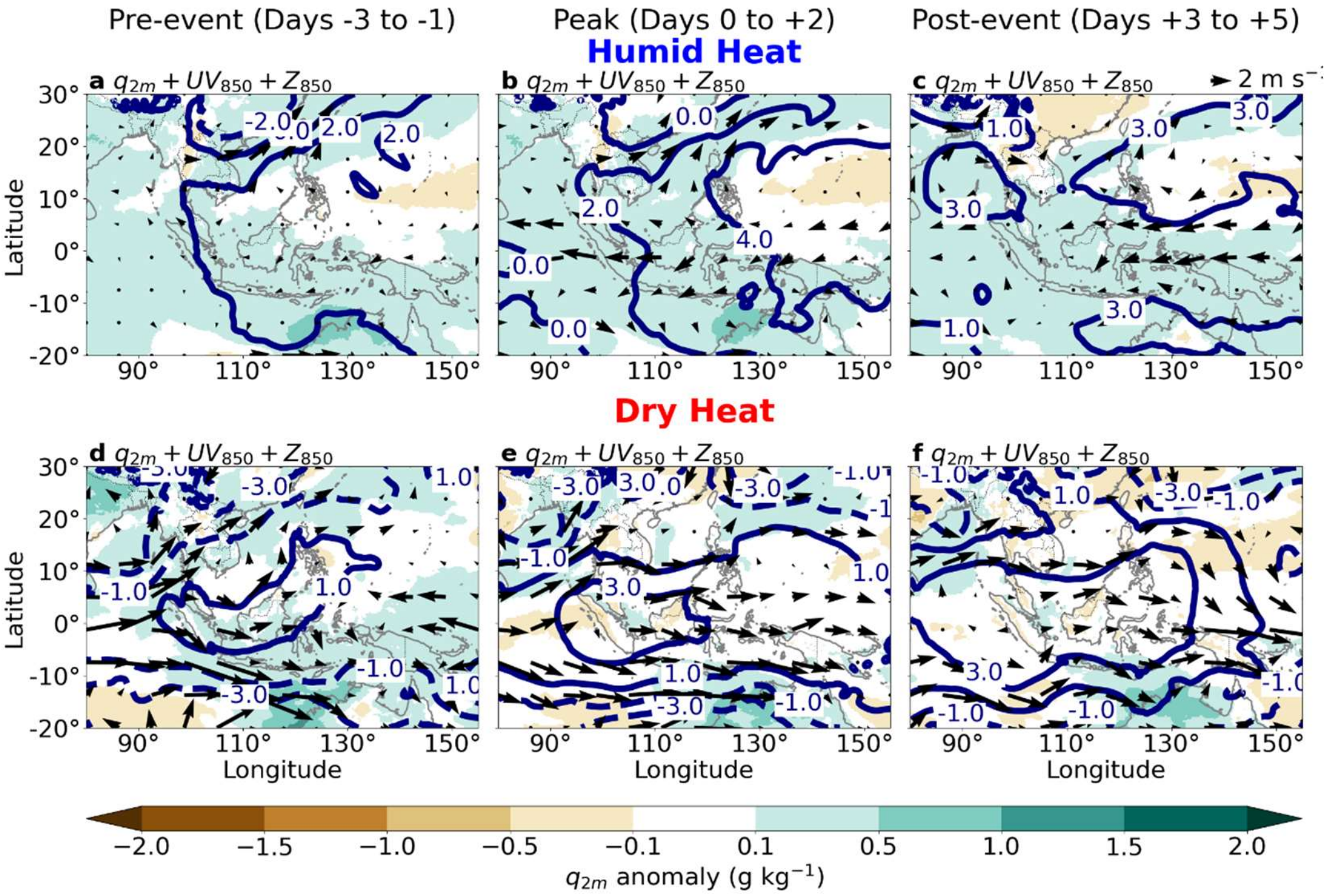

Supplementary Figure 2. Composite anomalies of specific humidity during HHE in the West. a-c Specific humidity anomalies (g kg^{-1}; shading) during pre-event (a), peak (b), and post-event (c). Overlaid are anomalies of 850 hPa geopotential height (m; contours) and wind (m s^{-1}; vectors). **d-f** As in a-c, but for DHEs. Shaded areas denote 95% confidence intervals. All anomalies are calculated relative to the local daily mean climatology over the 1993-2024.

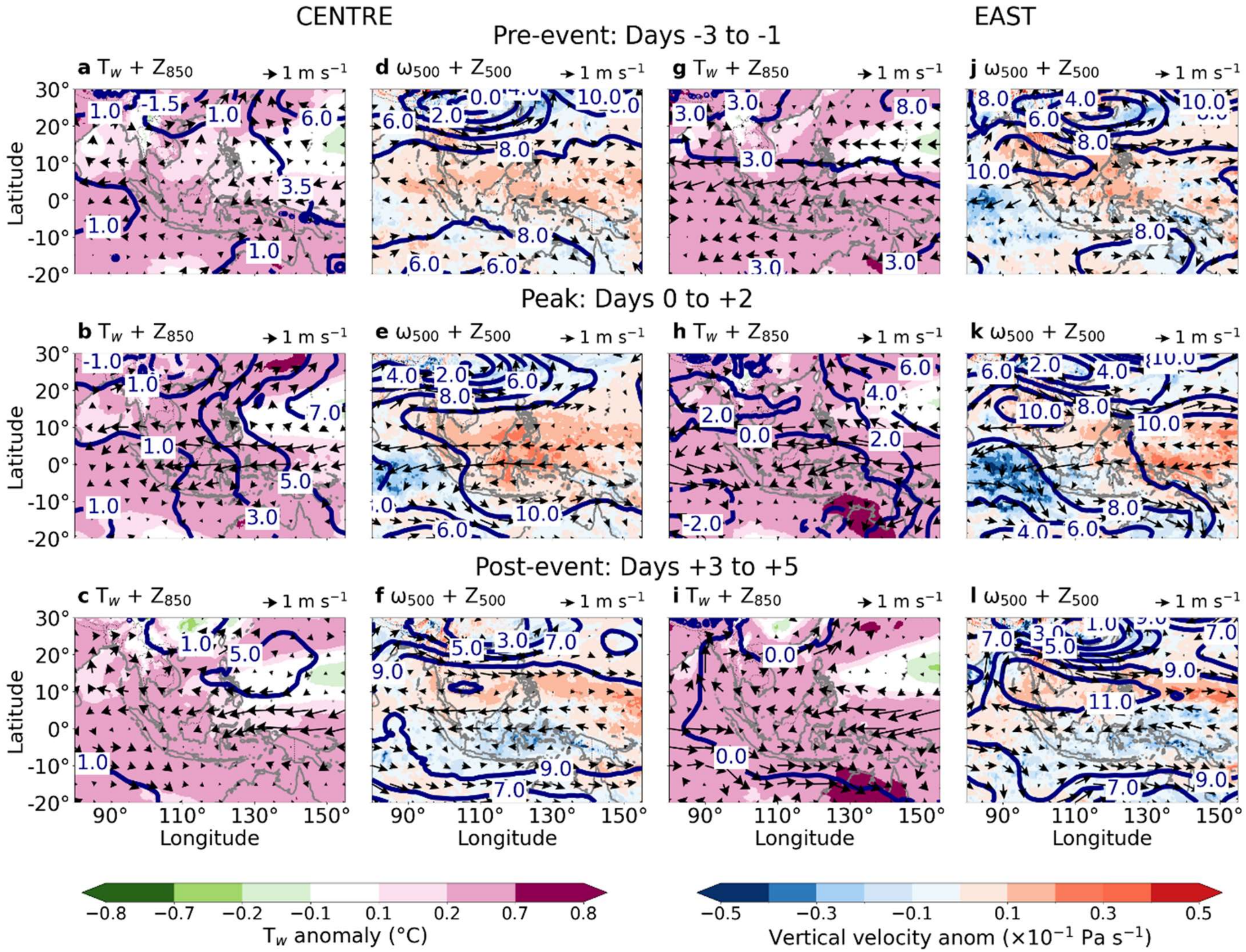

Supplementary Figure 3. Anomaly composites during pre-event (top), peak (middle), and post-event (bottom) period of the HHEs across the Centre and East regions. a,b,c Anomalies of 2-m T_w (°C; shading) relative to the local daily mean climatology, overlaid with anomalies of 850 hPa geopotential height (m, contours) and wind (m s^{-1}, vector) anomalies for the Centre region. **d,e,f** Anomalies of vertical velocity (ω) at 500 hPa (Pa s^{-1}; shading), overlaid with

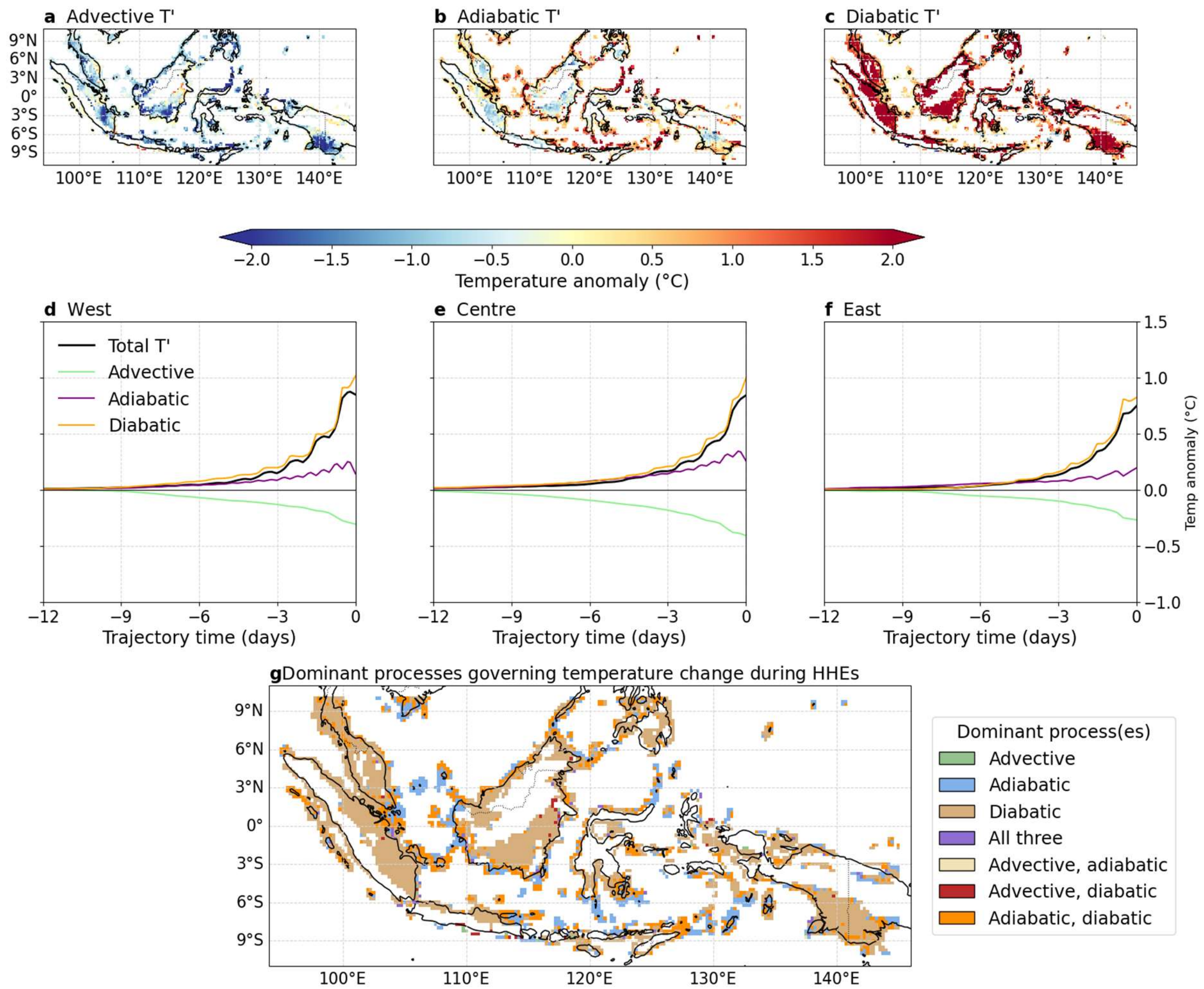

Supplementary Figure 4. Lagrangian decomposition of temperature anomalies during HHEs using ERA5 data from 1993-2024. a–c Spatial distribution of the individual contributions to the total temperature anomaly (T') at the onset of the HHE. The anomalies are decomposed into horizontal advection (a), adiabatic warming/cooling due to vertical motion (b) and diabatic heating/cooling (c). **d–f** Backward-trajectory evolution of the temperature budget for West (d), Centre (e), and East (f). The black line indicates the total T', while the coloured lines represent the cumulative contributions of advective (light green), adiabatic (purple), and diabatic (orange) processes along the paths of air parcels ending in the HHE occurrences at $t = 0$. **g** Dominant processes governing temperature change during HHEs based on the relative magnitude of the budget components. Grid cells are classified according to the process(es) that contribute most significantly to the local temperature anomaly. A process is considered "dominant" if its contribution is at least twice as large as the next largest positive contribution. Combined categories (e.g., "advective, adiabatic") indicate regions where multiple processes contribute substantially to the extreme temperature anomalies.

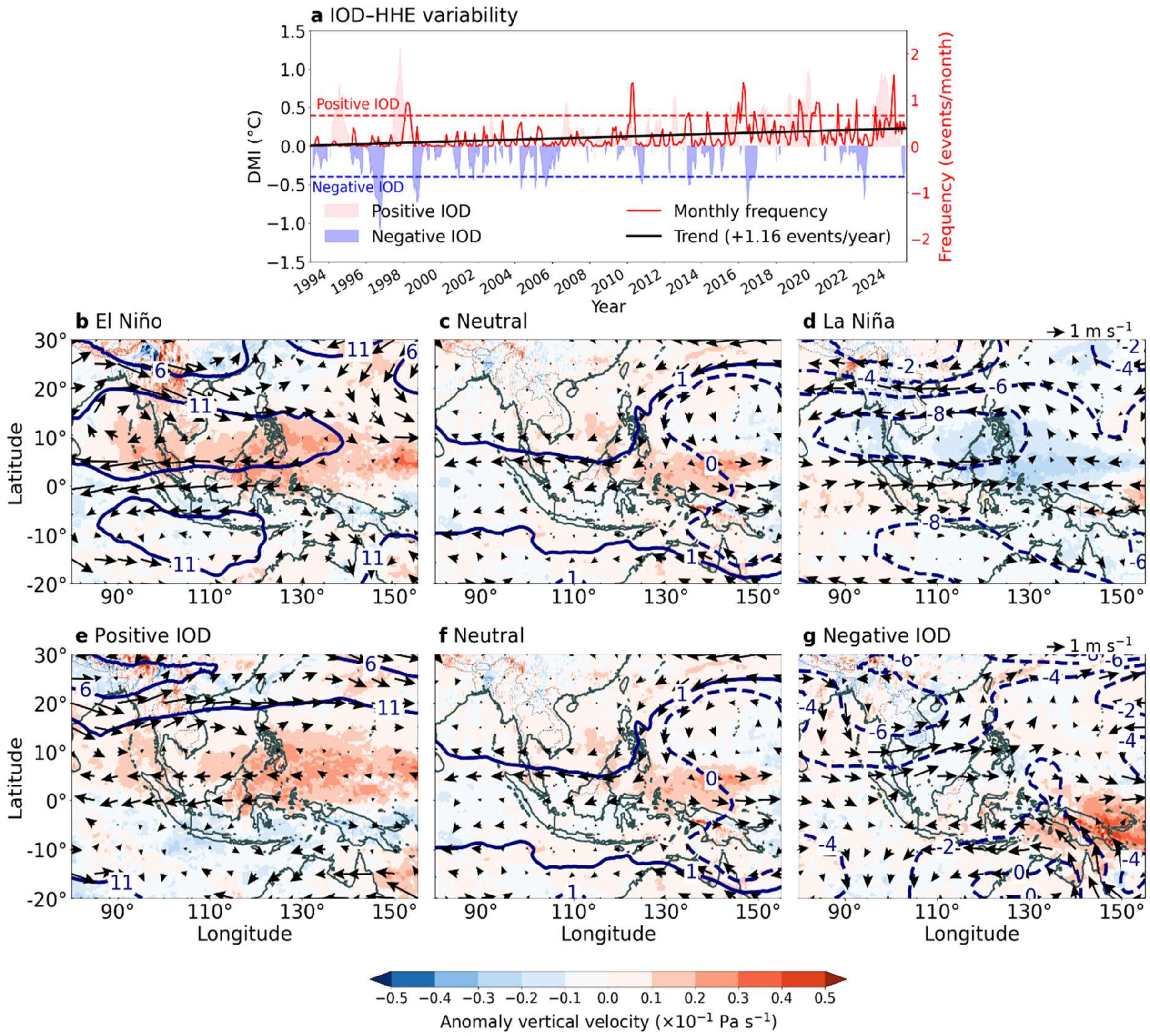

Supplementary Figure 5. HHE occurrence relative to IOD phase and 500hPa atmospheric circulation during ENSO, IOD and neutral years. a Positive SST anomalies (°C; red shading) indicates positive IOD, meanwhile negative anomalies (°C; blue shading) indicate negative IOD. Horizontal dashed lines denote thresholds for strong IOD (+0.4°C; red) and strong negative IOD events (−0.4°C; blue). The left y-axis represents SST anomalies over Indian ocean and the right y-axis shows the frequency of humid heat extremes (HHEs), calculated for each land grids. The solid red lines indicate monthly variability of HHE frequency from 1993-2024, and black dashed line shows its long-term trend. **b-d** Anomalies of 500 hPa vertical velocity (ω, Pa s^{-1}; shading), geopotential height (m; contours), and wind (m s^{-1}; vectors) during MAM season under El Niño (**b**), neutral (**c**), and La Niña (**d**) years. **e-f** same as b-d, but for positive IOD (**e**), neutral (f), and negative IOD (**g**) years. Positive *ω* anomalies correspond to anomalous downward motion (subsidence), while negative anomalies indicate anomalous upward motion (ascent). Anomalies are calculated relative to local climatological daily means for the 1993–2024 period, smoothed with a 15-day moving average.

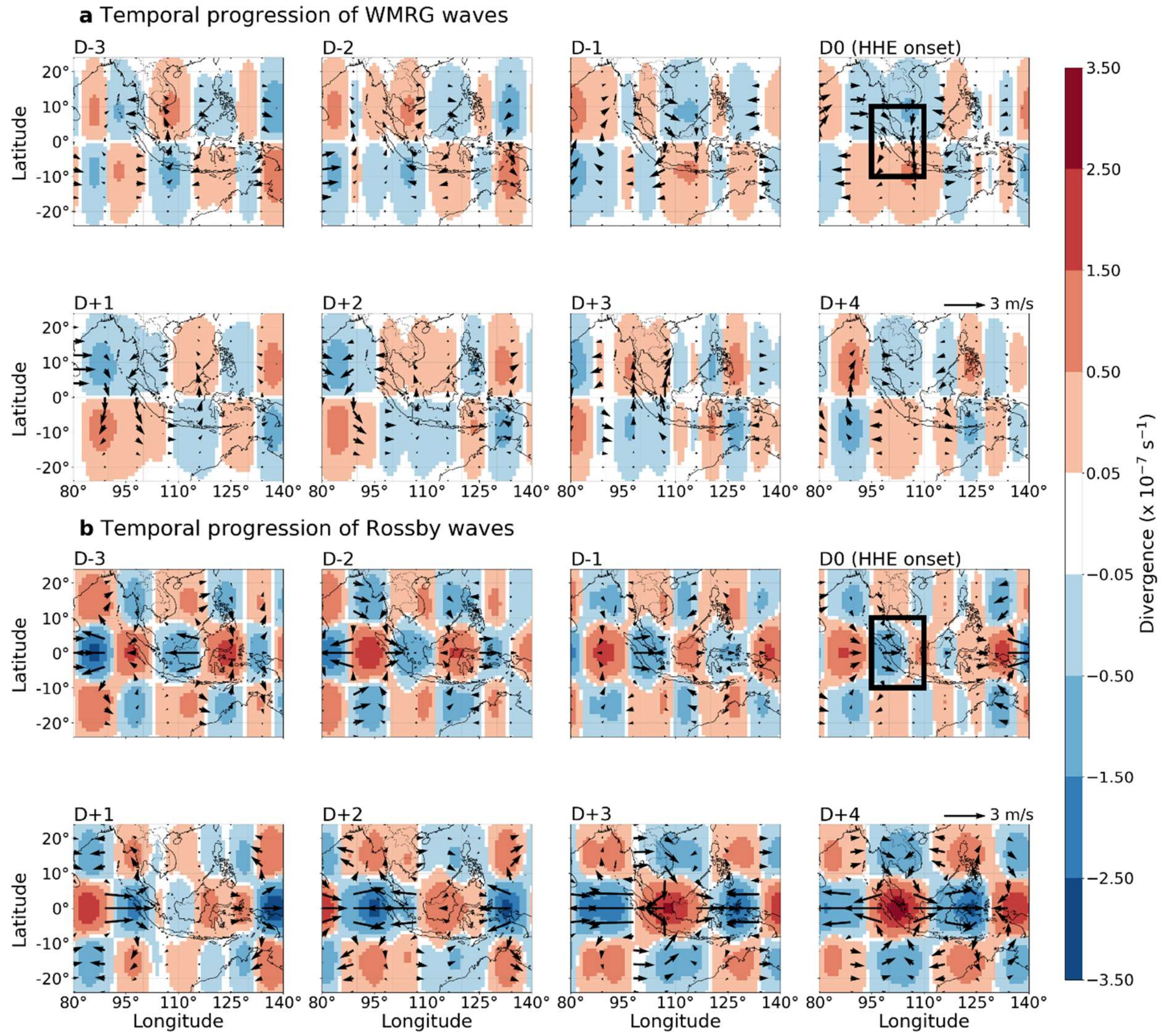

Supplementary Figure 6. Temporal progression of WMRG and Rossby waves during the pre-event, peak, and post-event of HHEs across the West region. a 850 divergence (s^{-1}; shading) and zonal wind ($m\ s^{-1}$) anomalies, from three days before (D-3) to four days after (D+4) the onset day (D0) of WMRG waves. **b** As in (a) but for Rossby waves. The black rectangle in the D0 panel represents the West region. Anomalies are calculated relative to 1993–2024 daily means and smoothed using a 15-day moving average.

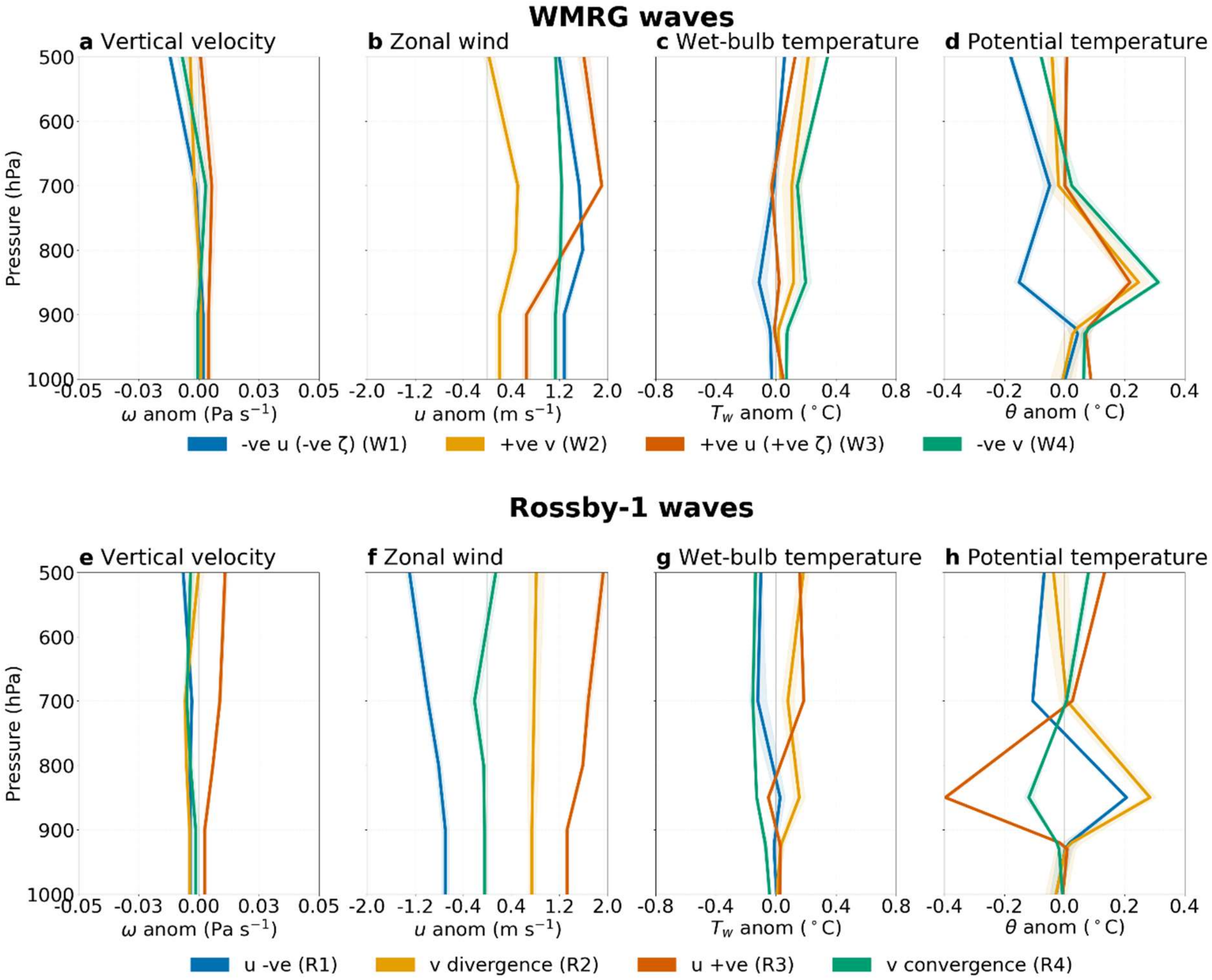

Supplementary Figure 7. Composites of the vertical atmospheric structure during active WMRG and Rossby wave phases. a Vertical wind (*ω,* Pa s^{-1}), **b** zonal wind (*u*, m s^{-1}), **c** T_w (°C), **d** potential temperature (*θ*, °C) anomalies for each WMRG phase. **e-h** As in panel (a-d), but for Rossby waves. The phases are labelled W1–W4 for WMRG and R1-R4 for Rossby waves, following the cycle defined by Crook et al.[23]: Anomalies are for MAM period relative to the 1993–2024 climatology, averaged over 10°S-10°N and 95°-109°E. Note that these composites represent all active WMRG and Rossby waves, not only those occurring during HHEs.

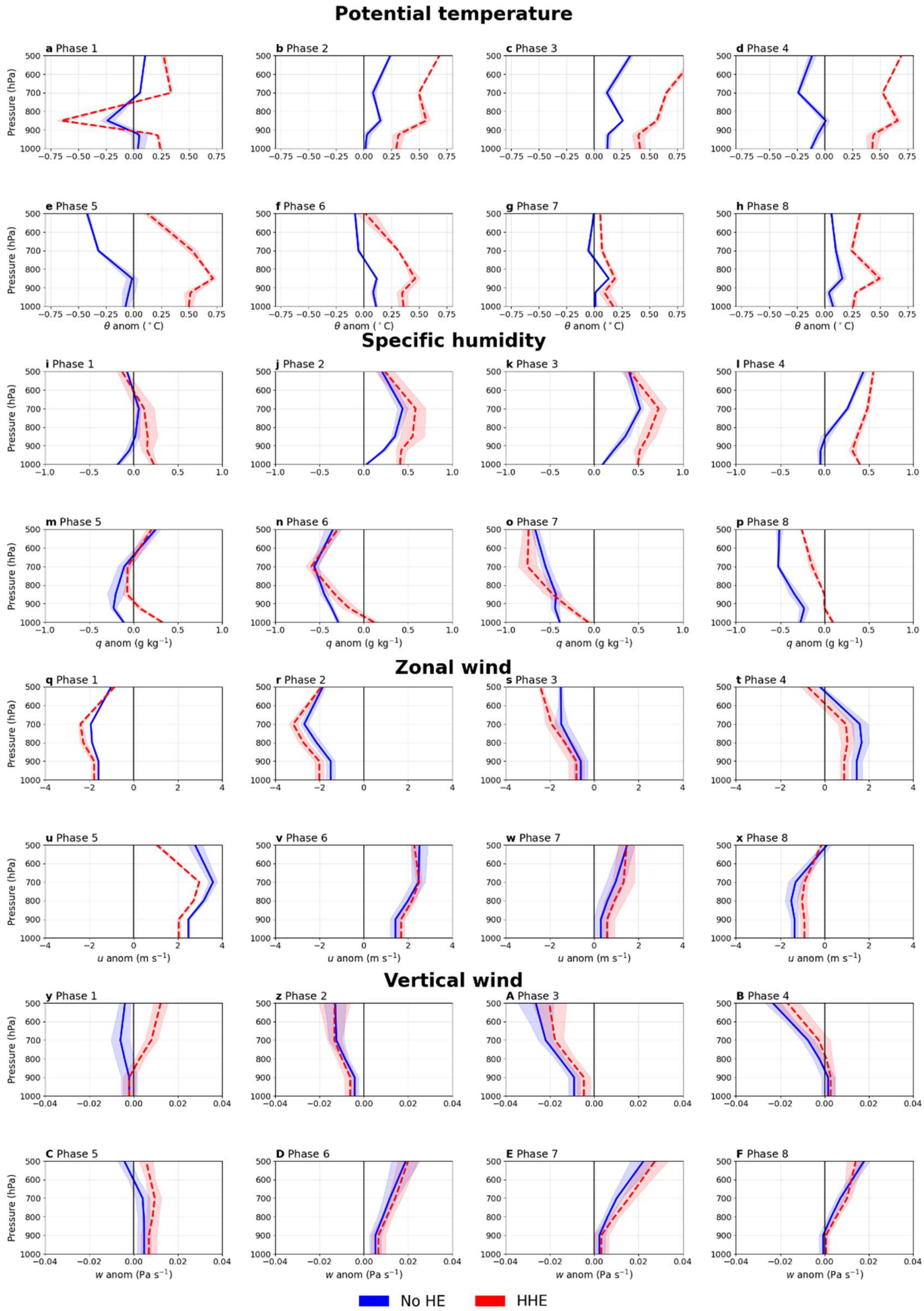

Supplementary Figure 8. Composite of the vertical atmospheric structure during non-HHE vs HHE days under different MJO phases. a-h Potential temperature (θ, °C), **i-p** specific humidity (q, g kg^{-1}), **q-x** zonal wind (u, m s^{-1}), **y-F** vertical wind (ω, Pa s^{-1}) anomalies from 1000 – 500 hPa for HHE (red) and non-HHE days (blue). Anomalies are for March–May relative to the 1993–2024 climatology, averaged over 10°S-10°N and 95°-140°E.

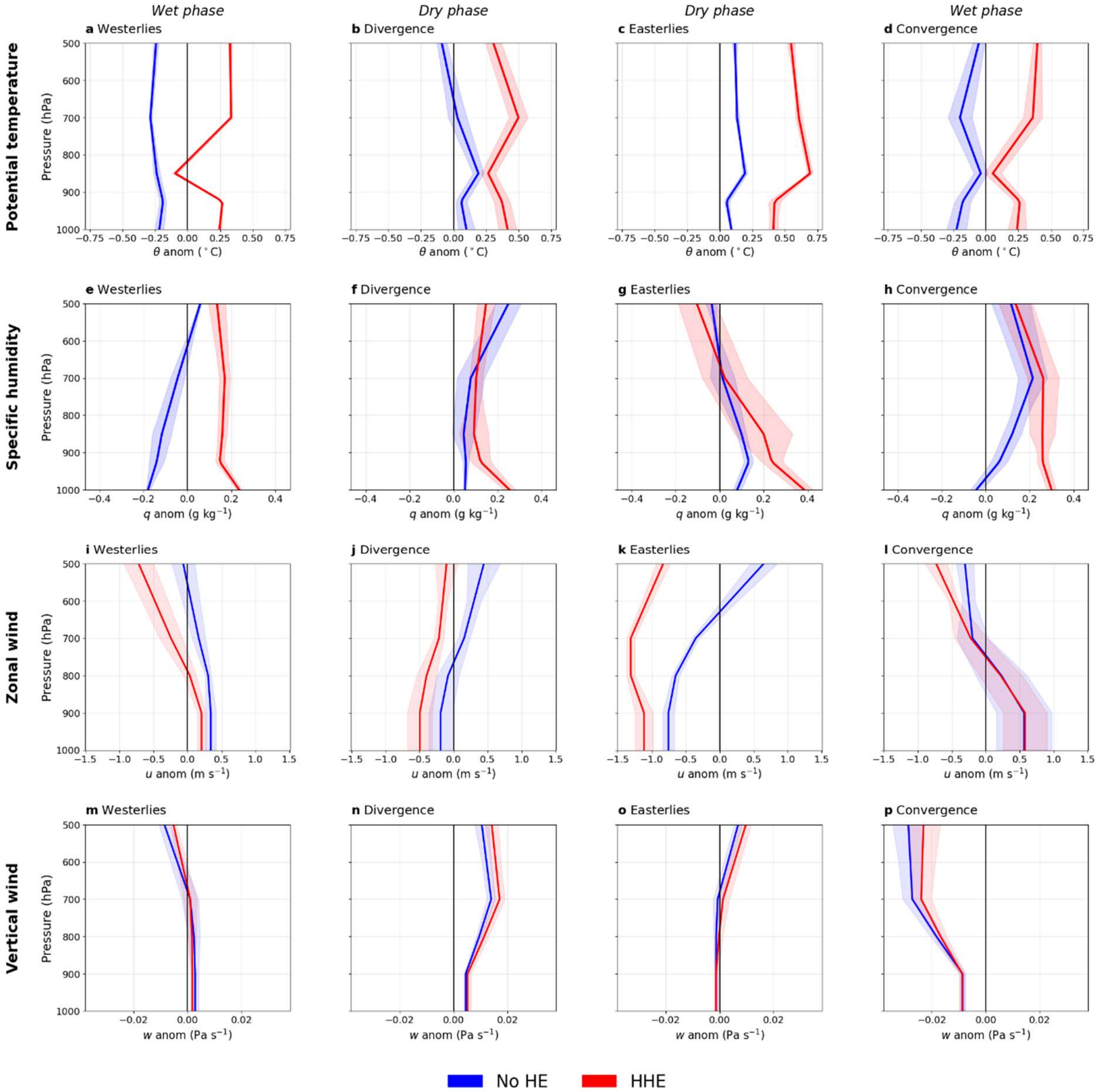

Supplementary Figure 9. Composite of the vertical atmospheric structure during non-HHE vs HHE days under different Kelvin wave phases. a-d Potential temperature (θ, °C), **e-h** specific humidity (q, g kg^{-1}), **i-l** zonal wind (u, m s^{-1}), **m-p** vertical wind (ω, Pa s^{-1}) anomalies from 1000 – 500 hPa for HHE (red) and non-HHE days (blue). Anomalies are for March–May relative to the 1993–2024 climatology, averaged over 10°S-10°N and 95°-140°E.

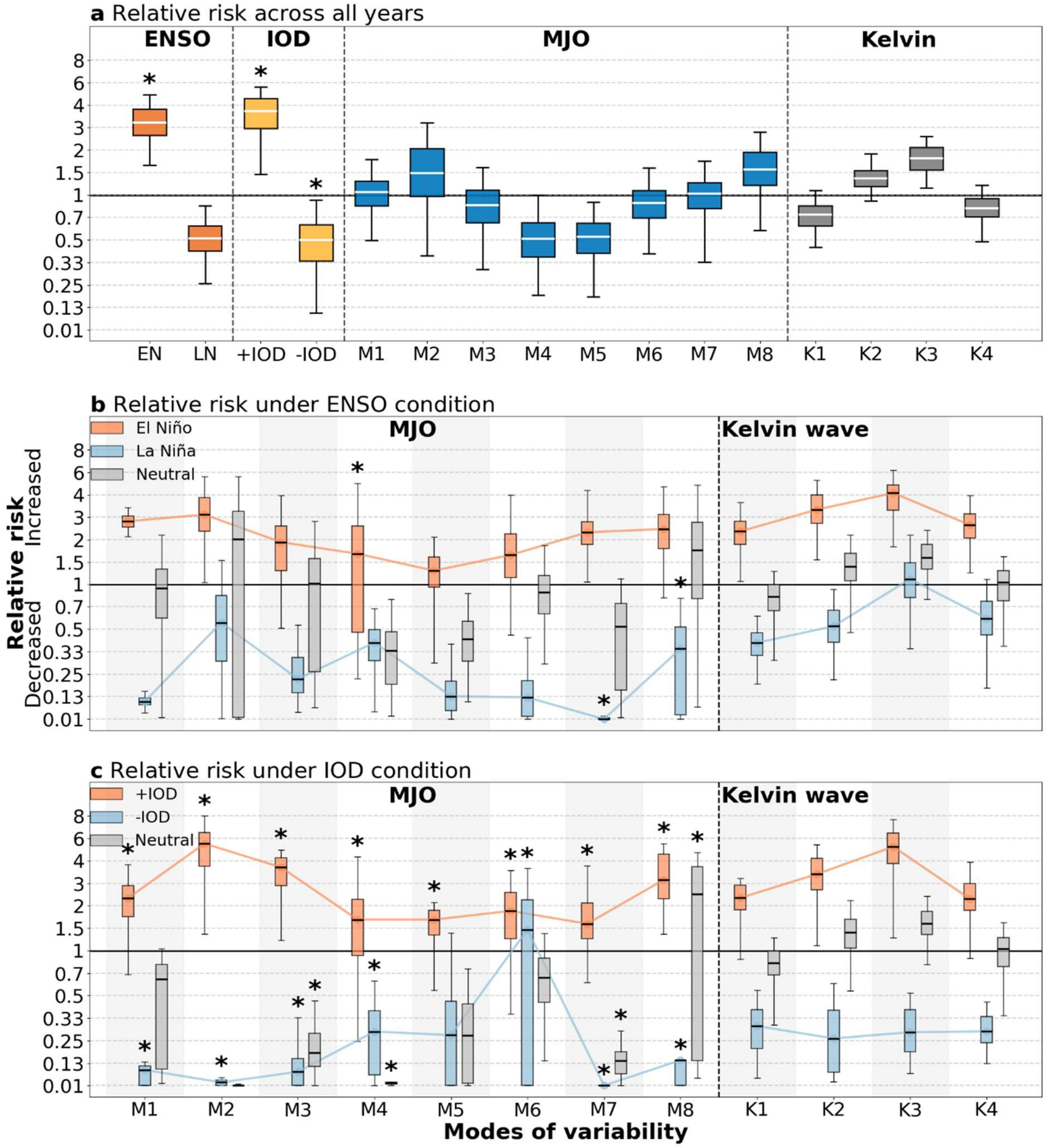

Supplementary Figure 10. Relative risk of HHE occurrences associated with modes of variability in the Centre region during MAM. a Relative risk of HHE occurrence during the active phases of each mode relative to that mode's neutral phase for the 1993-2024 period: ENSO (EN: El Niño, LN: La Niña), IOD (+IOD: s IOD, -IOD: negative IOD), MJO (M[number]: MJO in phase 1-8), and Kelvin waves (K1: westerlies, K2: divergence, K3: easterlies, K4: convergence). **b** Relative risk of MJO and Kelvin wave phases conditioned on ENSO state. **c** As in (b), but conditioned on IOD state. Orange (blue) lines connect the median relative risk of coupled MJO – Kelvin and El Niño - Positive IOD (La Niña – Negative IOD) phases. In all panels, the boxes show the interquartile range, the centre line represents the median, and the whiskers indicate the 2.5th and 97.5th percentiles of the bootstrap distribution. The asterisk (*) symbols above the upper whiskers represent a smaller sample size (≤10 active years for unconditioned interannual modes; (≤30 active days for intraseasonal and conditioned phases) for the corresponding composite. Relative risk values are calculated from the 32-year daily climatology from 1993–2024, with statistical uncertainty is assessed using 1000 bootstrap resamples within each active phase category.

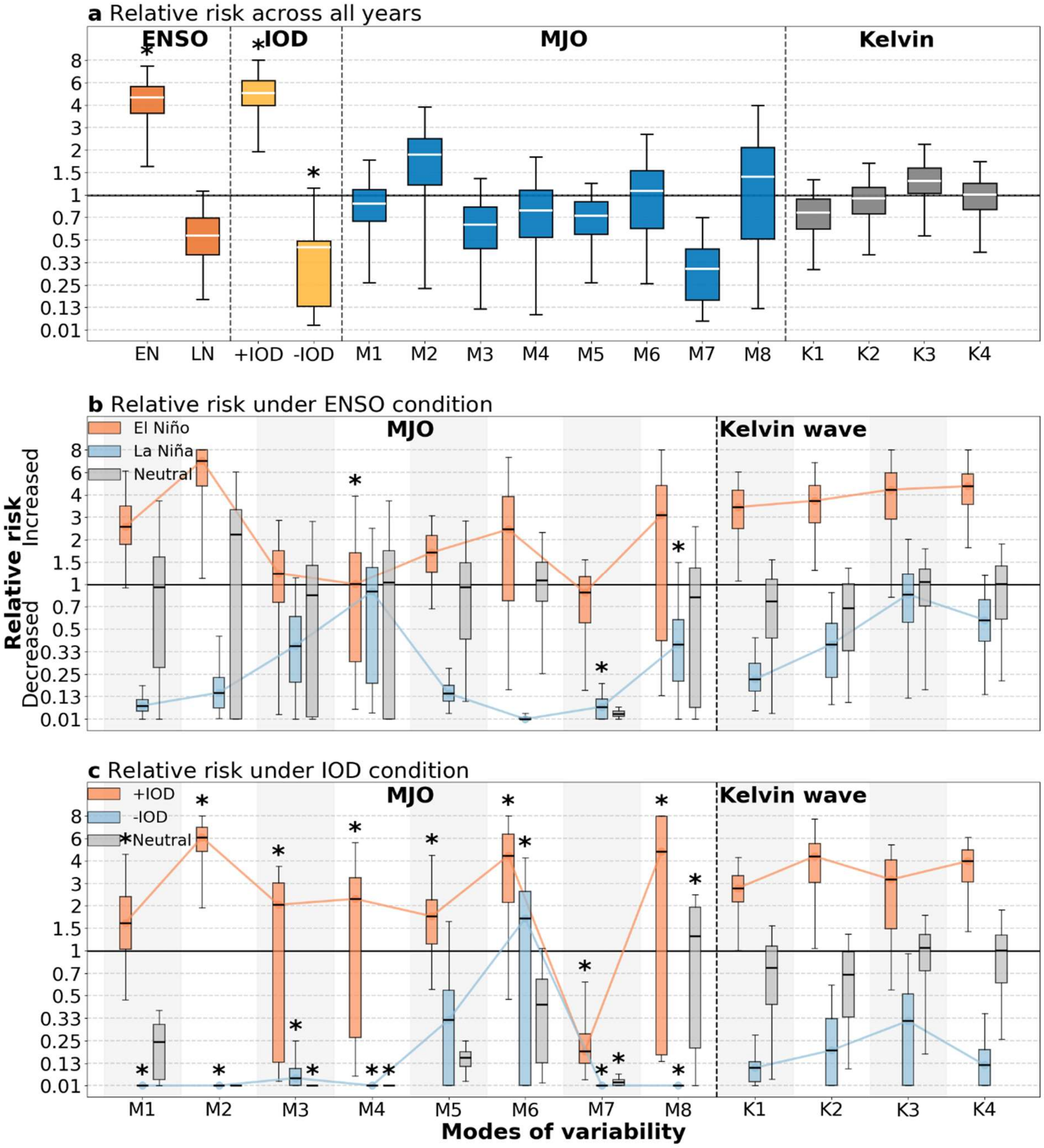

Supplementary Figure 11. Relative risk of HHE occurrences associated with modes of variability in the East region during MAM. a Relative risk of HHE occurrence during the active phases of each mode relative to that mode's neutral phase for the 1993-2024 period: ENSO (EN: El Niño, LN: La Niña), IOD (+IOD: s IOD, -IOD: negative IOD), MJO (M[number]: MJO in phase 1-8), and Kelvin waves (K1: westerlies, K2: divergence, K3: easterlies, K4: convergence). **b** Relative risk of MJO and Kelvin wave phases conditioned on ENSO state. **c** As in (b), but conditioned on IOD state. Orange (blue) lines connect the median relative risk of coupled MJO – Kelvin and El Niño - Positive IOD (La Niña – Negative IOD) phases. In all panels, the boxes show the interquartile range, the centre line represents the median, and the whiskers indicate the 2.5th and 97.5th percentiles of the bootstrap distribution. The asterisk (*) symbols above the upper whiskers represent a smaller sample size (≤10 active years for unconditioned interannual modes; (≤30 active days for intraseasonal and conditioned phases) for the corresponding composite. Relative risk values are calculated from the 32-year daily climatology from 1993–2024, with statistical uncertainty is assessed using 1000 bootstrap resamples within each active phase category.

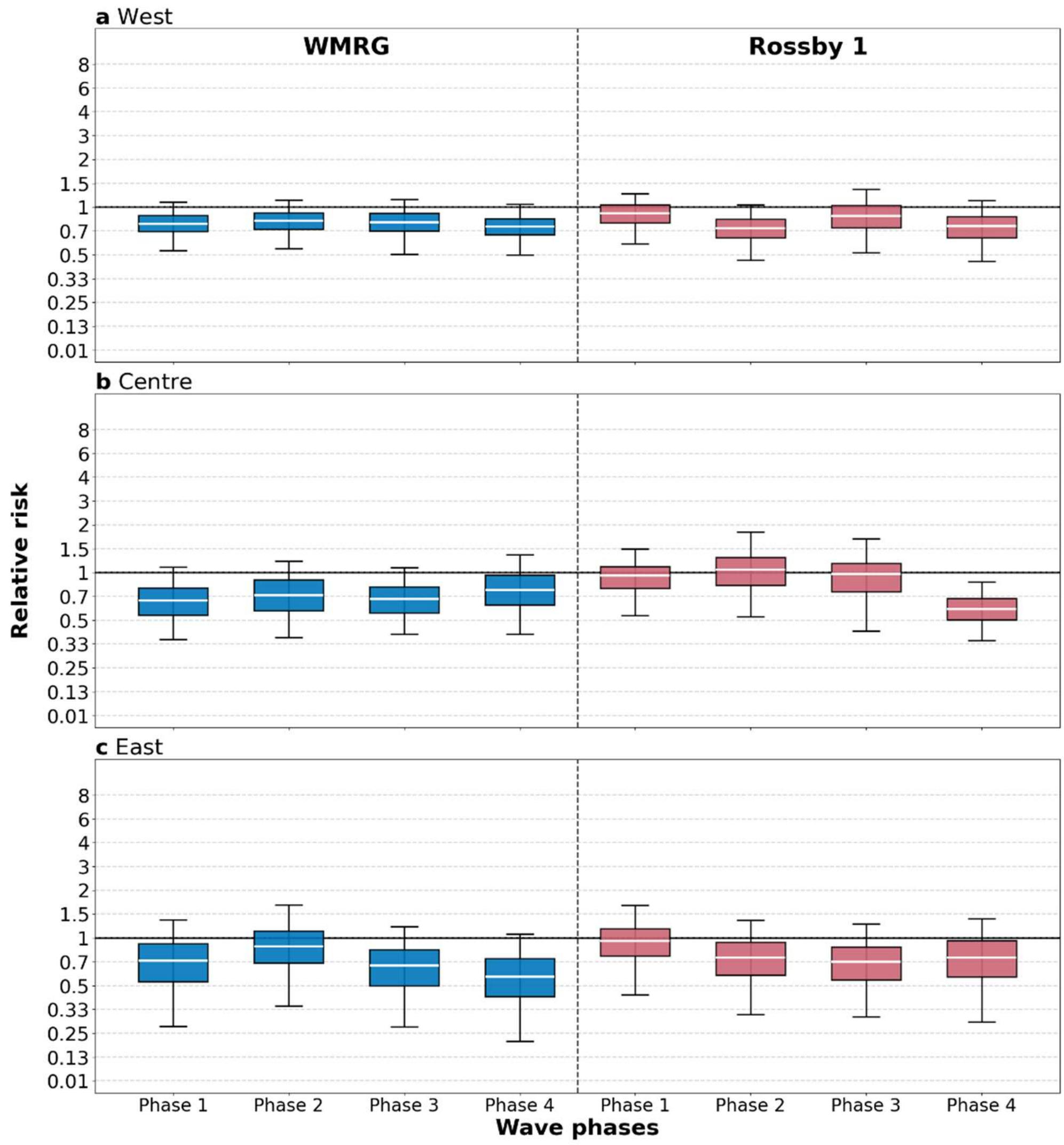

Supplementary Figure 12. Relative risk of HHE occurrences associated with WMRG and equatorial Rossby wave across equatorial SEA during MAM period. **a** Relative risk during the active phases of each mode relative to that mode's neutral phase: WMRG (Phase 1: positive vorticity (wet phase), Phase 2: northward *v* phase, Phase 3: Negative vorticity (dry phase), Phase 4: southward *v* phase) and equatorial Rossby wave (Phase 1: easterly phase (dry phase), Phase 2: *v* divergence phase (dry phase), Phase 3: Westerly phase (wet phase), Phase 4: *v* convergence phase (wet phase)), shown as bar plot. **b-c** As in a, but for the Centre (b) and East (c) regions. In all panels, the boxes show the interquartile range, the central white line represents the median, and the whiskers indicate the 2.5th and 97.5th percentiles of the bootstrap distribution. Relative risk values are calculated from the 32-year daily climatology from 1993–2024, with statistical uncertainty is assessed using 1000 bootstrap resamples within each active phase category.

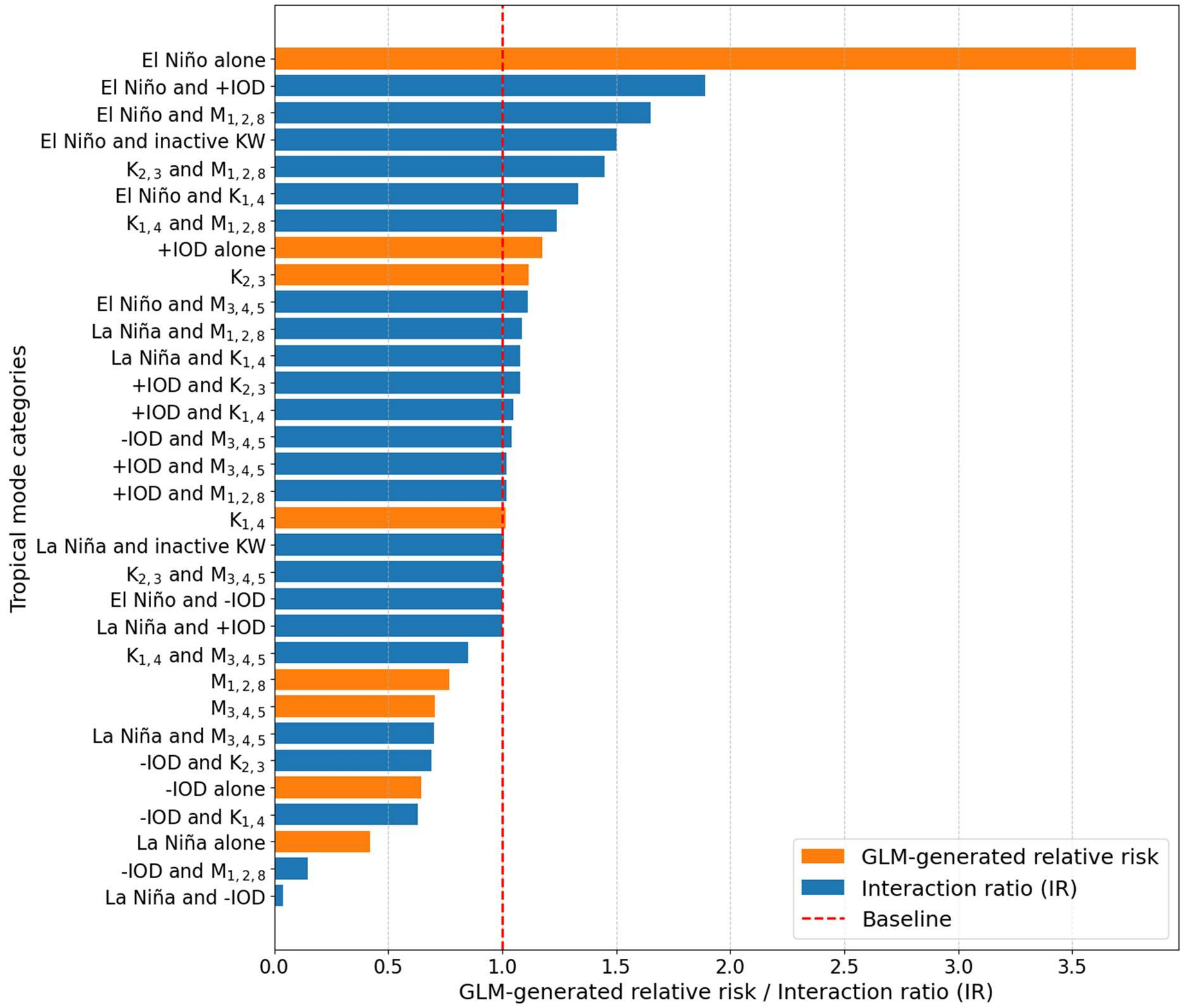

Supplementary Figure 13. Independent and interactive effects of tropical climate modes on HHEs quantified using negative binomial GLM. Orange bars show the isolated main effect of individual modes relative to a baseline (red dashed line). Blue bars display the Interaction Ratio (IR) when two modes co-occur. Values >1.0 indicate risk amplification, values < 1 indicate a damping effect, and values equal to 1 indicate no interaction on the multiplicative scale.

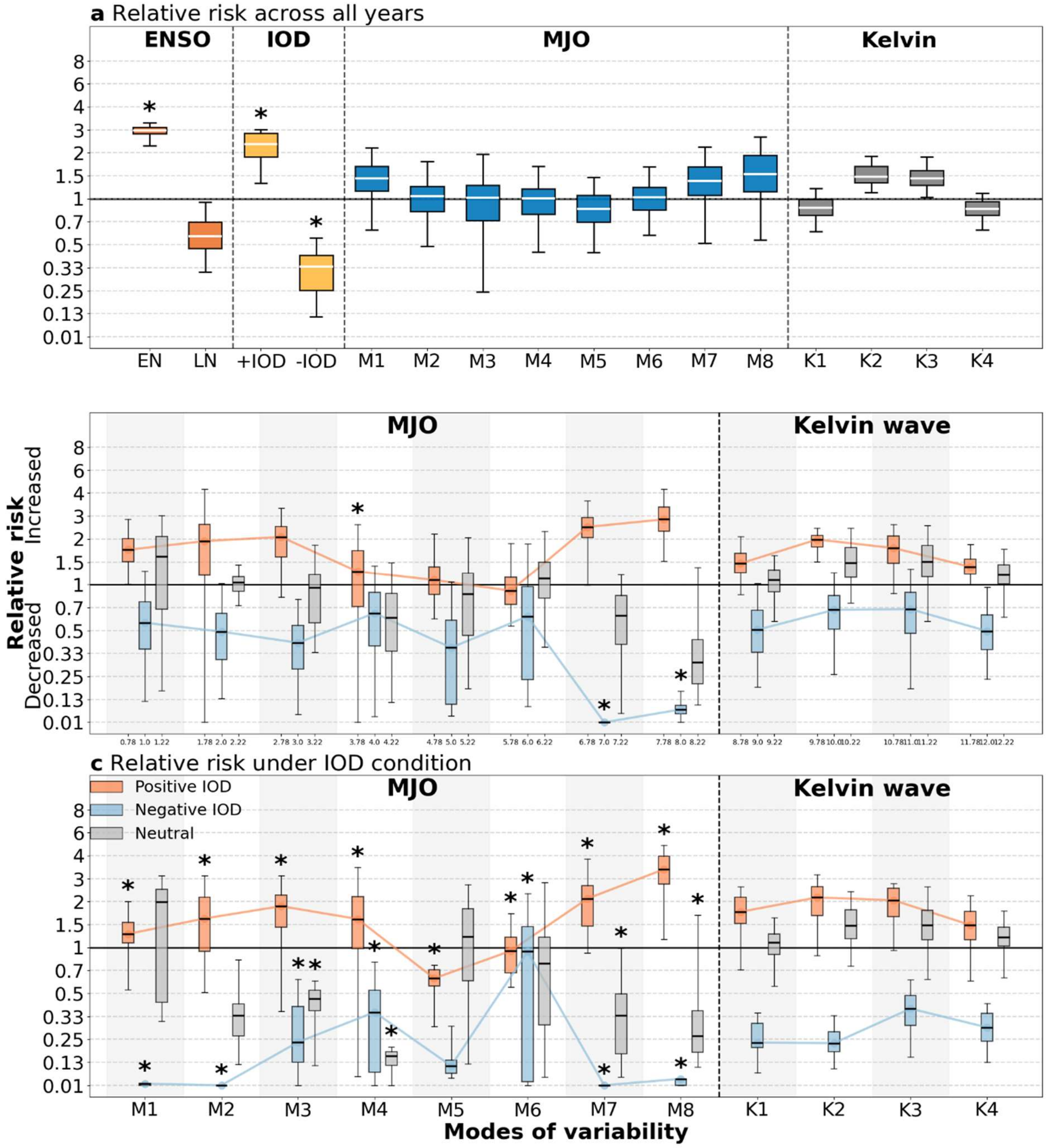

Supplementary Figure 14. Relative risk of DHE occurrences associated with modes of variability in the West region during MAM. a Relative risk of HE occurrence during the active phases of each mode relative to that mode's neutral phase for the 1993-2024 period: ENSO (EN: El Niño, LN: La Niña), IOD (+IOD: s IOD, -IOD: negative IOD), MJO (M[number]: MJO in phase 1-8), and Kelvin waves (K1: westerlies, K2: divergence, K3: easterlies, K4: convergence). **b** Relative risk of MJO and Kelvin wave phases conditioned on ENSO state. **c** As in (b), but conditioned on IOD state. Orange (blue) lines connect the median relative risk of coupled MJO – Kelvin and El Niño - Positive IOD (La Niña – Negative IOD) phases. In all panels, the boxes show the interquartile range, the centre line represents the median, and the whiskers indicate the 2.5th and 97.5th percentiles of the bootstrap distribution. The asterisk (*) symbols above the upper whiskers represent a smaller sample size (≤10 active years for unconditioned interannual modes; (≤30 active days for intraseasonal and conditioned phases) for the corresponding composite. Relative risk values are calculated from the 32-year daily climatology from 1993–2024, with statistical uncertainty is assessed using 1000 bootstrap resamples within each active phase category.

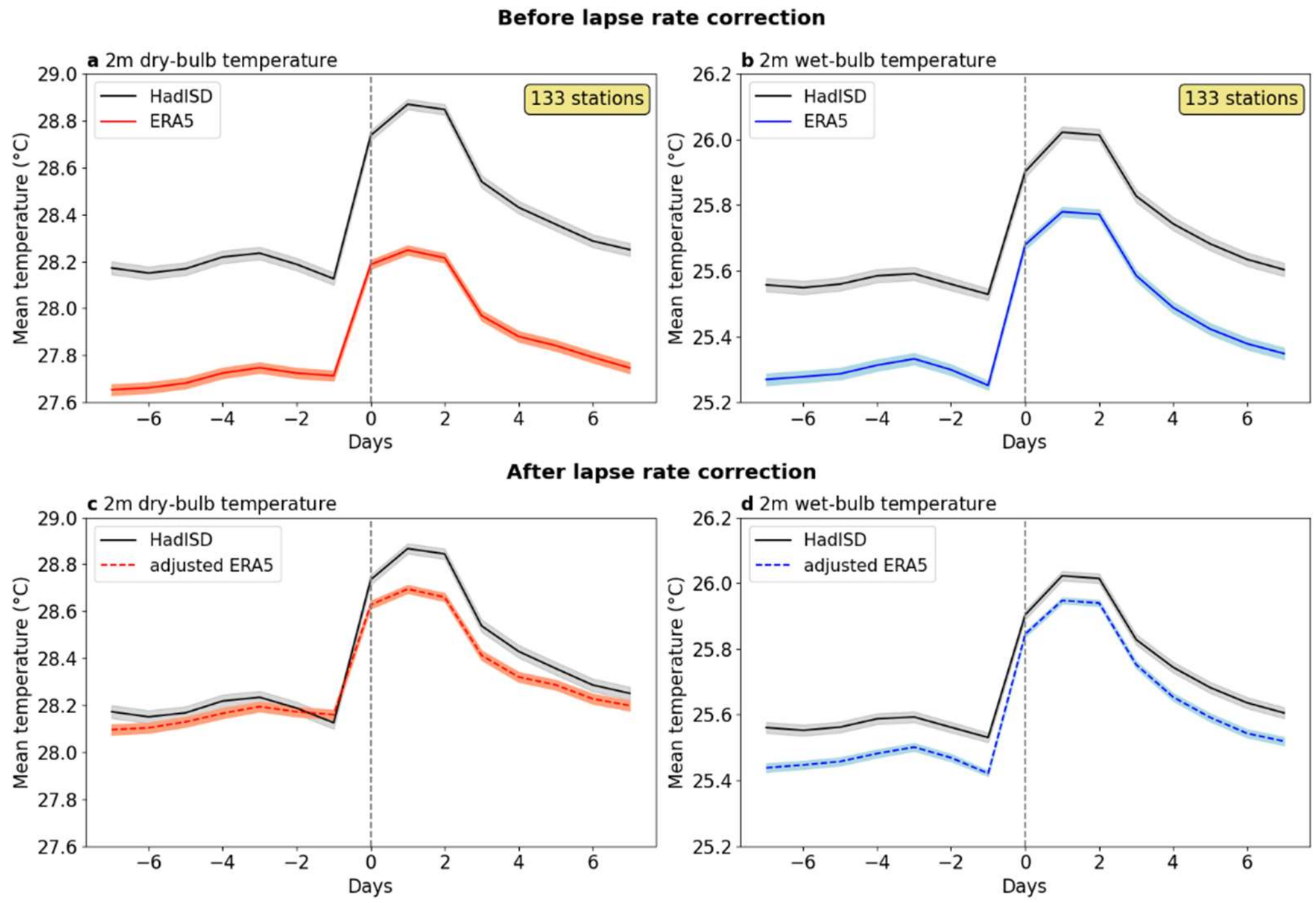

Supplementary Figure 15. Composite time series of daily mean variables. a,c T_{2m} (orange lines) during DHEs, **b,d** T_w (blue lines) during HHEs. Values are derived from selected HadISD stations and corresponding ERA5 grid cells across equatorial SEA (1993–2024) before (solid lines) and after (dashed-lines) lapse rate correction applied. T_{2m} are adjusted using the dry adiabatic lapse rate, while T_w are corrected using a physically derived moist adiabatic lapse rate. Time series span from 7 days prior to 7 days after the onset day (Day 0) of each heat extreme. The number of stations with sufficient data is indicated in the top right corner of each panel.

Supplementary Table 1. Summary of HHE event counts in the West region

Tropical modes	Active years	Active days	HHE grid-days	Median relative risk
**EN**	7	644	179772	3.8
**LN**	12	1104	41074	0.6
**+IOD**	2	184	29417	2.9
**-IOD**	3	276	10195	0.5
**M1**	24	246	26414	1.3
**M2**	24	220	23973	1.4
**M3**	25	114	11339	1.1
**M4**	20	141	7869	0.7
**M5**	27	325	22008	0.8
**M6**	28	211	14063	0.9
**M7**	22	117	9739	0.9
**M8**	18	111	10172	1.2
**K1**	30	1491	91671	1.0
**K2**	30	1155	108975	1.7
**K3**	30	722	80495	1.9
**K4**	30	1164	70864	1.1
		**Under El Niño-conditioned**		
**M1**	6	75	10282	2.2
**M2**	6	41	6128	2.2
**M3**	6	32	4441	1.9
**M4**	5	20	2064	1.4
**M5**	7	96	10920	1.5
**M6**	7	78	5873	1.1
**M7**	7	48	6704	2.1
**M8**	7	49	8306	2.2
**K1**	7	368	31283	2.1
**K2**	7	276	43556	3.3
**K3**	7	164	28743	3.8
**K4**	7	277	21127	1.8
		**Under La Niña-conditioned**		
**M1**	10	96	3598	0.6
**M2**	9	89	2544	0.5
**M3**	10	42	721	0.3
**M4**	8	64	2293	0.6
**M5**	12	124	2076	0.2
**M6**	11	56	103	0.05
**M7**	8	30	0	0
**M8**	2	26	94	0.1
**K1**	12	544	11255	0.6
**K2**	12	436	14614	0.8
**K3**	12	279	12856	1.1
**K4**	12	429	10496	0.7
		**Under Neutral ENSO-conditioned**		
**M1**	8	75	7534	1.3
**M2**	9	90	5301	1.1
**M3**	9	40	2877	1.0
**M4**	7	57	2512	0.5
**M5**	8	105	7012	1.0
**M6**	10	77	6087	1.3
**M7**	7	39	1035	0.3
**M8**	5	36	1772	0.8
**K1**	11	579	23133	0.9
**K2**	11	443	32805	1.6
**Tropical modes**	**Active years**	**Active days**	**HHE grid-days**	**Median relative risk**
**K3**	11	279	30896	2.5

**K4**	11	458	21241	1.5
		**Under positive IOD-conditioned**		
**M1**	2	21	2137	1.8
**M2**	2	28	5164	3.2
**M3**	2	9	2656	3.6
**M4**	2	16	908	0.8
**M5**	2	18	1357	1.0
**M6**	2	20	1680	1.1
**M7**	2	16	807	0.8
**M8**	1	3	488	1.8
**K1**	2	126	10813	2.5
**K2**	2	92	10085	3.4
**K3**	2	59	7317	3.7
**K4**	2	101	7233	2.1
		**Under negative IOD-conditioned**		
**M1**	2	9	0	0
**M2**	2	17	0	0
**M3**	2	5	22	0.05
**M4**	2	15	729	0.6
**M5**	2	31	2224	1
**M6**	2	15	1421	1.8
**M7**	1	2	0	0
**M8**	1	14	334	0.4
**K1**	3	154	2265	0.4
**K2**	3	122	3616	0.8
**K3**	3	71	1467	0.6
**K4**	3	115	1687	0.6
		**Under neutral IOD -conditioned**		
**M1**	4	45	4397	1.5
**M2**	5	45	137	0.03
**M3**	5	26	199	0.2
**M4**	3	26	275	0.2
**M5**	4	56	2031	0.7
**M6**	6	42	2986	0.8
**M7**	4	21	28	0.05
**M8**	3	19	1050	1.1
**K1**	6	299	9055	0.9
**K2**	6	229	13104	1.6
**K3**	6	149	13112	2.5
**K4**	6	242	11321	1.5
